# Development and validation of a carnitine cycle and transport disorders (CCD) panel: an ONT-compatible multi-gene diagnostic kit for newborn and selective screening

**DOI:** 10.1186/s13023-025-03775-4

**Published:** 2025-05-26

**Authors:** Gökçe Akan, Mehmet Cihan Balcı, Gülten Tuncel, Meryem Karaca, Hasan Hüseyin Kazan, Ahmet Çağlar Özketen, Özge Özgen, Gülden Fatma Gökçay, Fatmahan Atalar

**Affiliations:** 1 DESAM Institute, Near East University, Mersin 10, Türkiye; 2 Division of Pediatric Nutrition and Metabolism, Istanbul Medical Faculty, Istanbul University, Istanbul, Türkiye; 3 Department of Medical Biology, Gulhane Faculty of Medicine, University of Health Sciences, Ankara, Türkiye; 4 Rare Diseases Research Laboratory, Istanbul Medical Faculty, Istanbul University, Istanbul, Türkiye; 5 Department of Rare Diseases, Child Health Institute, Istanbul University, Istanbul, Türkiye

**Keywords:** Carnitine transport and cycle disorders, Third generation sequencing, Oxford nanopore technologies, Genetic diagnosis, Multi–gene diagnostic kit, CCD panel, *In Silico* analysis

## Abstract

Carnitine transport and cycle disorders (CCD) are a group of metabolic disorders characterized by either carnitine depletion or dysfunction in the carnitine cycle, a critical process for the transport of fatty acids into the mitochondria and their subsequent β-oxidation. Clinically, CCD can manifest with a wide range of symptoms, including hypoketotic hypoglycemia, which may be accompanied by signs of liver dysfunction, hepatic steatosis, myopathy and cardiomyopathy. Biochemical diagnosis typically involves measuring carnitine and acylcarnitine levels in blood, alongside organic acid profiling in urine. However, due to phenotypic overlaps with other metabolic disorders, precise molecular diagnosis is essential for accurate disease classification and subtype determination. The present study aimed to develop and clinically validate a novel CCD panel, specifically designed for Oxford Nanopore Technologies (ONT) platform compatibility. The panel targeted four key CCD related genes (*CPT-1*, *CPT-2*, *SLC22A5* and *SLC25A20)*. An amplification-based library preparation method pooling 21 primers specific to the CCD-related genes into two tubes was optimized. The panel was then applied to screen 20 patients previously diagnosed with CCD via second-generation sequencing platform. Comparative analysis of results from both platforms revealed a 100% concordance in detecting pathogenic, likely pathogenic, and variants of unknown significance associated with CCD. In silico analysis was also performed to predict the pathogenic potential of the variants of unknown significance. Here we report the development and clinical validation of a multi-gene diagnostic panel for ONT platform. The results demonstrated the feasibility of ONT-based genetic testing for CCD and set the stage for the development of similar diagnostic panels for other genetic disorders, offering a streamlined and putatively cost-effective alternative to current sequencing methodologies.

**Supplementary Information:**

The online version contains supplementary material available at 10.1186/s13023-025-03775-4.

## Introduction

**Fig. 1:**
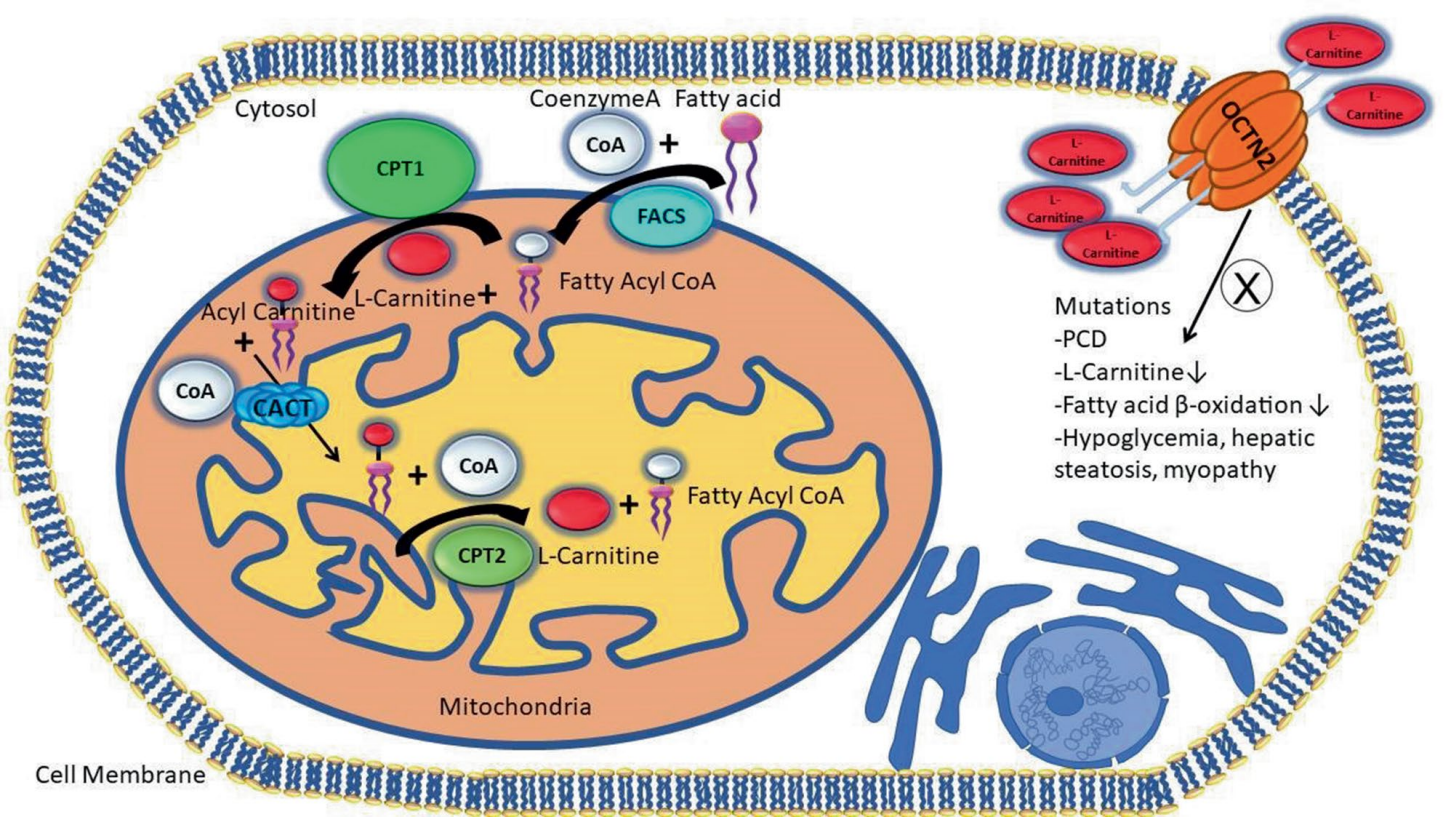
Schematic representation of L-carnitine transport and its function in fatty acid β-oxidation. OCTN2: Organic cation/carnitine transporter; FACS: Acyl-CoA synthetase; CPT1: carnitine palmitoyl transferase 1; CPT2: carnitine palmitoyl transferase 2; CACT: carnitine acylcarnitine translocase

Carnitine (3-hydroxy-4-N-trimethylaminobutyrate) is essential for β-oxidation of fatty acids, facilitating the transport of long-chain fatty acids to mitochondria via the carnitine shuttle. Carnitine depletion, whether intracellularly or in plasma, disrupts the mitochondrial carnitine-acyl carnitine cycle, leading to symptoms ranging from mild fatigue to severe cardiac complications [1–3]. Carnitine deficiency is categorized into two subtypes: primary carnitine deficiency (PCD) also known organic cation/carnitine transporter 2 (OCTN2) deficiency and secondary carnitine deficiency (SCD) [4]. Diagnosis involves measuring serum carnitine levels. Carnitine levels below 20 µmol/l indicate carnitine deficiency and require genetic screening to confirm mutations in related genes for differential diagnosis [5]. PCD, an autosomal recessive disorder linked to solute carrier family 22 member 5 (*SLC22A5*; *OCTN2*) mutations, typically manifests in children aged 1–7 years, with a frequency of 1–5 per 10.000 and causes metabolic derangements including hypoketotic hypoglycemia, hepatic steatosis, myopathy, and potentially sudden death due to cardiomyopathy [4, 6]. The malfunction of OCTN2 leads to improper carnitine transport (Fig. 1), resulting in urine excretion and reduced plasma levels, impairing fatty acid oxidation [7]. In contrast, SCD is more common, milder and often associated with liver or kidney disease or medications like valproic acid [4, 8–9]. Carnitine biosynthesis and renal reabsorption maintain normal levels, making deficiency from inadequate intake rare [10]. Mitochondrial enzymes, in the carnitine cycle including carnitine palmitoyltransferase 1 and 2 (CPT-1 and CPT-2) regulate the trans-esterification of acyl groups of CoA to carnitine [11]. In the first step, acyl groups are transferred from CoA to carnitine by outer membrane bounded enzymes CPT-1a and CPT-1b. Subsequently, with the assistance of carnitine-acylcarnitine translocase (CACT), they undergo translocation across a specific region of the inner mitochondrial membrane (Fig. 1). Within the mitochondrial matrix, CPT-2 catalyzes the transfer of acyl groups to mitochondrial CoA, releasing free carnitine and generating acyl-CoA substrates for fatty acid oxidation. CACT and CPT-2 work together as a complex to facilitate the transfer of acyl-carnitine from the carrier to the enzyme in the inner mitochondrial membrane. Following the reaction, the released carnitine is transported back to the cytosol via the acyl-carnitine and carnitine antiport system. Proper fatty acid oxidation is essential, and many tissues rely on these carnitine-related reactions to meet their energy needs, making the functionality of CPT-1 and CPT-2 enzymes pivotal for efficient fatty acid oxidation. The critical role of CACT in energy metabolism is underscored by the discovery of autosomal recessive mutations in the *SLC25A20* gene, located at 3p21.31, which comprises nine exons. These mutations lead to CACT deficiency (OMIM # 212138), a severe mitochondrial fatty acid oxidation disorder that manifests early in life due to impaired β-oxidation [12, 13]. Mutations in *SLC25A20* gene prevent acyl-carnitines from reaching the mitochondrial matrix, significantly disrupting β-oxidation. This condition is more severe than PCD, which results from defects in the OCTN2 transporter (encoded by *SLC22A5*) [12].

Given the severity of carnitine transport and cycle disorders (CCD), early diagnosis and treatment are crucial. Many countries have implemented newborn screening programs, typically using tandem mass spectrometry (MS) on blood samples from the newborns to determine carnitine and acylcarnitine levels. However, due to the low specificity of free carnitine (C0) measurement in conventional newborn screening (NBS), additional diagnostic evaluations are often required to identify missed and/or confirmed suspected PCD cases. These evaluations may include urine organic acid analysis, clinical examination, and family history assessment, all of which support differential diagnosis beyond the scope of standard NBS framework [14, 15]. These evaluations may include organic acid analysis in urine, clinical examination, and family history assessment, which aid in differential diagnosis not classified within the NBS framework. When there is suspicion of defects in carnitine transport or the cycle, genetic analysis is carried out to confirm the diagnosis. If molecular testing is inconclusive or reveals variants of unknown significance, an alternative option is skin biopsy to assess carnitine transport in cultured fibroblasts [16].

Next-generation sequencing (NGS) provides significant benefits over traditional methods, particularly through its high-throughput and parallel analysis of extensive nucleic acid samples. NGS is cost-effective, efficient, and robust, making it ideal for comprehensive genome or exome profiling across large patient cohorts [17, 18]. Various NGS platforms, such as Illumina, Pacific Biosciences, and Oxford Nanopore Technology (ONT), utilize different methodologies, including fluorescently labeled nucleotides and pore-based electrical currents, to improve sequencing accuracy and read length [19]. ONT is notable for its long-read sequencing, which enables detailed analysis of structural variants and DNA methylation patterns. Combining short- and long-read sequencing enhances accuracy [20, 21], and targeted ONT sequencing has been successfully applied in streamlined newborn screening, as demonstrated in detecting biotinidase deficiency (BTD) [22].

The primary aim of this study is the methodological development and clinical validation of a novel, ONT-compatible multi-gene diagnostic panel; the CCD panel, for the rapid and accurate detection of pathogenic variants in *SLC22A5*, *CPT-1*, *CPT-2*, and *SLC25A20* genes. To achieve comprehensive coverage, we have designed a multiplex long-range PCR-based assay that targets both coding, untranslated regions (UTR) and intronic regions that harbor reported pathogenic (P) and likely pathogenic (LP) variants of these genes. This panel is designed for application within the context of NBS, with the primary aim of confirming or further elucidating alterations in the acylcarnitine profile indicative of PCD, CPT1, CPT2, or CACT deficiencies. Although certain metabolic disorders, such as CoA synthesis defects, may present with overlapping biochemical signatures, this targeted molecular approach facilitates precise confirmation in a cost-effective and time-efficient manner. As a secondary objective, we performed variant interpretation using in silico tools to assess the pathogenicity of variants of unknown significance (VUS) and LP variants. Together, these efforts are intended to establish a streamlined, cost-effective, and scalable platform for the genetic diagnosis of CCD, with significant implications for both newborn and selective screening programs.

## Materials and methods

### Research design

A newborn screening test panel targeting CCD was developed, incorporating four relevant genes associated with these conditions. The panel was initially validated through laboratory-based assays and subsequently tested for clinical utility. To assess its diagnostic performance, the ONT-based CCD panel efficacy was applied to a cohort of patients previously diagnosed using Illumina-based sequencing, allowing for a direct comparative validation of two sequencing platforms. The study cohort comprised patients admitted to the Department of Pediatrics, Division of Nutrition and Metabolism, Istanbul Medical Faculty, Istanbul University, between 2021 and 2023.

### Study cohorts

This study involved a total of 20 patients with CCD who were undergoing treatment at Department of Pediatrics, Division of Nutrition and Metabolism clinic at Istanbul Medical Faculty. The demographic and clinical characteristics of the patients are summarized in Table 1. The CCD-diagnosed cohort consisted of 9 male and 11 female patients, aged between 10 days and 48 years old (3 neonates, 12 children under 10 years and 5 adults). Additionally, 13 patients had consanguineous parents. The clinical diagnoses of CCDs were based on the results of carnitine level assessments using the tandem MS spectrometry. The PCD (OCTN2 deficiency) was diagnosed based on low levels of free carnitine (C0). CPT-1 and CPT-2 deficiency were diagnosed using the following diagnostic ratios: C0/(C16 + C18), (C16 + C18:1) /C2, respectively (Table 1) [23].

**Table 1:** Demographic, clinical and biochemical data of patients with carnitine transport and cycle disorders

Patients	Sex	Age	Consanguinity	Diagnosis	C0 (Free Carnitine (µmol/l)	C16 (µmol/l)	C18 (µmol/l)	C18:1 (µmol/l)	C2 (µmol/l)	CPT1 (C0/(C16 + C18))	CPT2 ((C16 + C18:1)/C2)
1	M	6.5 y	2nd cousins	CPT-1 Deficiency	263.00	0.10	0.16	0.11	32.30	1011.54	< 0.01
2	M	4 y	1st cousins	CPT-1 Deficiency	255.78	0.04	0.07	0.01	28.51	2325,27	< 0.01
3	F	22 m	No	CPT-1 Deficiency	169.00	0.10	0.06	0.07	28.30	1056.25	< 0.01
4	M	10 d	1st degree cousins	CPT-1 Deficiency	144.00	0.11	0.14	0.04	24.80	576.00	< 0.01
5	F	5 years	1st degree cousins	CPT-1 Deficiency	147.00	0.06	0.03	0.03	39.80	1633,00	< 0.01
6	M	3 years	1st degree cousins	CPT-2 Deficiency	18,70	2.06	1.47	1.32	5.91	5.30	0.57
7	F	18 years	1st degree cousins	CPT-2 Deficiency	24.40	0.02	0.85	0.71	7.53	28.05	0.09
8	M	18 years	ND	CPT-2 Deficiency	27.60	1.40	1.58	1.66	6.91	9.26	0.44
9	F	48 years	1st degree cousins	CPT-2 Deficiency	12.40	0.82	0.86	0.97	5.54	7.38	0.32
10	F	45 years	3rd degree cousins	CPT-2 Deficiency	17.70	1.05	0.47	1.28	14.38	11.64	0.16
11	M	32 years	No	CPT-2 Deficiency	36.00	0.80	0.50	0.74	15.65	27.70	0.09
12	M	10.5 months	3rd degree cousins	CPT-2 Deficiency	NA	NA	NA	NA	NA	NA	NA
13	M	7 years	2nd degree cousins	CPT-2 Deficiency	37.00	3.11	1.41	1.72	17.35	8.18	0.27
14	F	21 months	No	PCD (OCTN2 Deficiency)	8.10	0.15	0.11	0.12	5.04	31.15	0.05
15	F	5 years	2nd degree cousins	PCD (OCTN2 Deficiency)	0.40	0.03	0.04	0.02	4.01	5.71	0.01
16	F	16 d	No	PCD (OCTN2 Deficiency)	11.10	0.53	0.46	0.52	15.2	11.21	0.07
17	F	8 years	ND	PCD (OCTN2 Deficiency)	11.70	0.22	0.12	0.20	1.6	34.41	0.26
18	F	2 years	2nd degree cousins	PCD (OCTN2 Deficiency)	1.20	0.05	0.04	0.07	5.49	13.33	0.21
19	F	45 d	1st degree cousins	PCD (OCTN2 Deficiency)	17.20	0.25	0.31	0.39	17.20	30.71	0.04
20	M	8	ND	PCD (OCTN2 Deficiency)	6.18	0.35	0.26	0.36	7.02	10.13	0.11

The table presents the quantitative analysis of free carnitine and acylcarnitines, assessed using MS/MS to provide insights into metabolic abnormalities and potential biomarkers in the patient cohort. NA: Not available (reported to be normal), M: male, F: female, y: years, m: months d: days, CPT-1: carnitine palmitoyltransferase I, CPT-2: carnitine palmitoyltransferase II, PCD: primary carnitine deficiency and OCTN2: organic cation/carnitine transporter 2, C0: free carnitine, C2: acetylcarnitine, C16: palmitoylcarnitine, C18: stearoylcarnitine, C18:1: oleoylcarnitine, CPT1 Ratio: C0 / (C16 + C18); CPT2 Ratio: (C16 + C18:1) / C2

In addition to these biochemical methods, the cohort underwent exome sequencing using an Illumina-based clinical exome sequencing platform (NextSeq 2000 platform; Illumina Inc., USA) to confirm genetic etiology of CCD prior to validation with the ONT-based CCD panel for validation [24]. The study cohort comprised 5 cases of carnitine palmitoyltransferase I deficiency (*CPT-1*), 8 cases of carnitine palmitoyltransferase II deficiency (*CPT-2*), and 7 cases of PCD (OCTN2 deficiency). Prior to participating in the study, written consent was obtained from each patient or legal guardian and ethical approval was obtained from the Istanbul Medical Faculty Ethics Committee (23.11.2022–1397546). All procedures in this study were performed in accordance with the current Helsinki Declaration.

### Genomic DNA preparation from whole blood

Two to five milliliters of whole blood were collected from each participant into an ethylenediaminetetraacetic acid (EDTA)-containing blood collection tubes and DNA extraction was performed with the QIAmp DNA Blood Mini kit (Qiagen, Hilden, Germany) according to the manufacturer’s protocol. All DNA samples were quantified using a Qubit 4 Fluorometer (Thermo Fisher Scientific, USA).

### Primer design

**Fig. 2:**
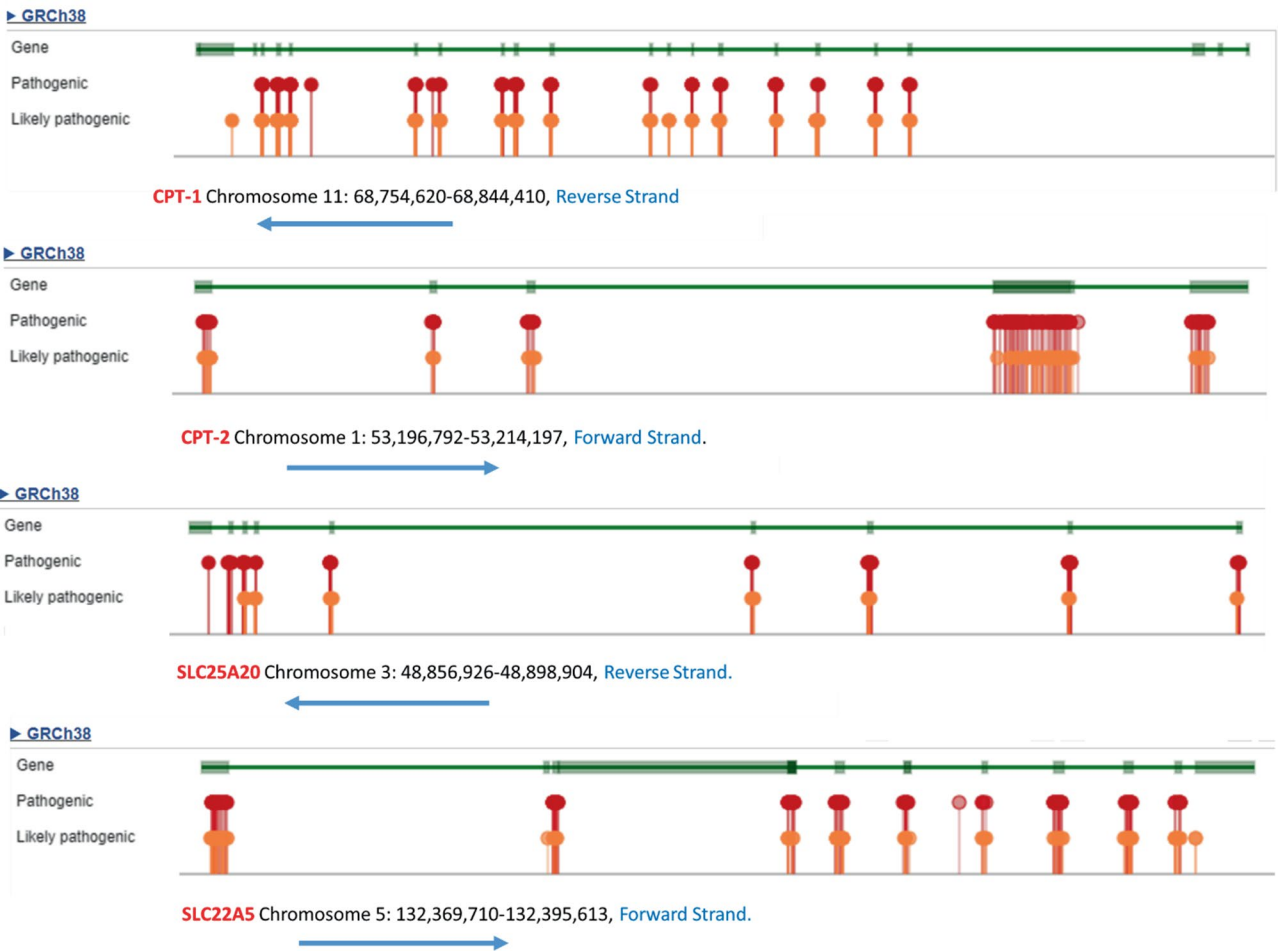
Graphical view of the locations of the pathogenic and likely pathogenic variants in the *CPT-1*,* CPT-2*,* SLC25A20* and *SLC22A5* genes. Adapted from *ClinVar* database (https://www.ncbi.nlm.nih.gov/clinvar/).

The *SLC22A5* (NM_003060.4) gene consists of 10 exons and harbors 318 pathogenic and likely pathogenic variations reported in the ClinVar database (https://www.ncbi.nlm.nih.gov/clinvar). The *CPT-1* (NM_001876.4) gene is composed of 19 exons and has 186 pathogenic and likely pathogenic variations; the *CPT-2* (NM_000098.3) gene includes 5 exons, with 243 pathogenic and likely pathogenic variations; the *SLC25A20* (NM_000387.6) gene is composed of 9 exons and a total of 72 variations were reported as pathogenic and likely pathogenic in the ClinVar database (Fig. 2).

**Fig. 3:**
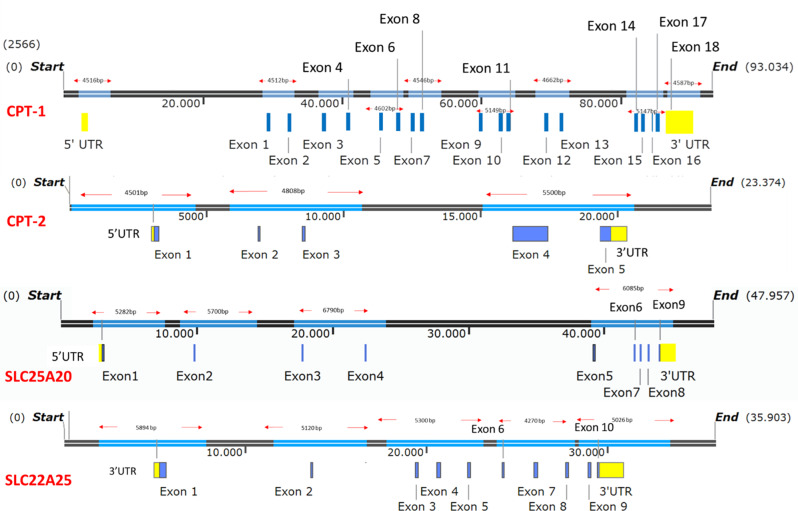
Graphical view of the locations of the designed primers covering the 5’UTR, all exons, 3’UTR, and relevant intronic regions of the *CPT-1*,* CPT-2*,* SLC25A20* and *SLC22A5* genes. The figure highlights the positions of the reported pathogenic and likely pathogenic variants as listed in the *ClinVar* database (https://www.ncbi.nlm.nih.gov/clinvar/)

To ensure comprehensive detection of pathogenic variants, a total of 21 long-range PCR primers were designed using NCBI Primer-BLAST to amplify key genomic regions of these genes [25]. The amplicon sizes ranged from 4 to 7 kb, enabling complete coverage of all clinically relevant regions, including coding exons, untranslated regions (5’ UTR, and 3’ UTR), and exon-intron boundaries. The design minimized off-target amplification and ensured the inclusion of splice site mutations, as well as intronic regions known to harbor pathogenic and likely pathogenic variants reported in the ClinVar database (Fig. 3). The 5’UTR was specifically included to capture potential regulatory variants that may contribute to clinical heterogeneity. Overall, the panel was strategically designed to prioritize regions with established pathogenicity, thereby ensuring high diagnostic accuracy while maintaining sequencing efficiency.

### Target amplification

Each of the 21 designed primer pairs was optimized by monoplex long polymerase chain reaction (PCR) with human reference DNA (Agilent Technologies, USA). A PCR mix was prepared by adding 30 ng of genomic DNA and 0.1 µM of each primer to a 2X Hi-Fi enzyme master mix (MobiomX, Türkiye). The thermal cycling conditions included an initial 3-minute denaturation step at 95 °C, followed by 40 cycles of denaturation (15 sec at 95 °C), annealing (45 Sec. 69 °C), and extension (4 min at 72 °C). A final extension step was performed at 72 °for 10 min. The PCR products were analyzed by electrophoresis on a 1% agarose gel to confirm the presence of the amplified DNA fragments. Following successful amplification by monoplex PCR, two distinct pools containing the 21 optimized primer pairs were used for multiplex PCR, maintaining a final primer concentration of 0.1µM. The existence of amplicons was verified by electrophoresis of the multiplex PCR products on a 1% agarose gel. After sequencing the amplicons generated from the human reference DNA, the primer concentrations were further optimized. The final optimized primer concentrations were then applied to sequence the patient samples.

### Library construction and nanopore sequencing

Library preparation was performed according to the manufacturer’s instructions using the SQK-NBD114.96 kits. Sequencing tethers and adapters were ligated to the amplicons, making the library ready for sequencing. Sequencing was carried out using the Mk1C device and FLO-MIN114 flow cell (Oxford Nanopore Technologies, Oxford, UK). Amplicons were first purified using the NEBNext Ultra II End-Repair/dA-Tailing Module (New England Biolabs, USA) and PCR products were subsequently purified using AMPure XP beads (Beckman Coulter Life Sciences, USA) following the supplier’s instructions. After repair, sample-specific barcodes were applied to the final products. Following purification, Adapter Mix II was used for pooling and ligating the barcoded products. Concentrations were determined using a Qubit 4 Fluorometer (Thermo Scientific, USA) after final purification. A total of 400 ng of the prepared library was loaded into the flow cell, and sequencing was conducted with the MinION Mk1c instrument over a 24-hour run.

### Data analysis

After ONT sequencing, raw Fastq file generated by ONT platform was processed. Accordingly, FastQC (http://www.bioinformatics.babraham.ac.uk/projects/fastqc/) was used for the quality assessment. The sequencing reads were aligned to the reference human genome (GRCh38) using Twist Exome 2.0 bed file (Twist Biosciences, USA) with MiniMap2 [26] and alignments were corrected by Medaka (https://github.com/nanoporetech/medaka). Variant calling was performed using Clair3-Trio [27], generating variant call format (VCF) files. For variant annotation, ANNOVAR [28] was used and variant filtering was carried out with VarAFT software [29]. The variants were filtered based on a frequency of less than 0.01% in common databases. Variant prioritization was carried out using ClinVar [30], Franklin by Genoox (https://franklin.genoox.com) and VarSome [31]. The unreported variants were classified according to the American College of Medical Genetics (ACMG) criteria [32]. For heterozygous variants, allele frequencies between 20 and 70% were considered, while allele frequencies greater than 70% were classified as homozygous.

### In silico analyses

The pathogenicity of the VUS and likely pathogenic LP variants, which were not previously reported in the literature and detected in the patients, was assessed through in silico bioinformatic tools. Protein structures and the domain specific-functional localizations were obtained using Protter [33] (https://wlab.ethz.ch/protter/). Visual representations were generated using UniProt [34] identifiers P50416, P23786 and O76082 for CPT-1, CPT-2 and OCTN2, respectively. Pathogenicity predictions for missense variants were conducted using MutPred2 [35] (http://mutpred.mutdb.org/index.html) and Missense3D [36] (https://missense3d.bc.ic.ac.uk/~missense3d/). For MutPred2 analysis, amino acid sequences in FASTA format were retrieved from NCBI Protein database [37]. Missense3D utilized AlphaFold models [38] of the proteins obtained from UniProt database. Indel VUS or LP variants were modeled using SWISS-MODEL [39] (https://swissmodel.expasy.org/) and 3D structures of the wild-type and mutant proteins were compared using Pairwise Structure Alignment tool of Protein Data Bank (PDB) [40].

## Results

### Panel optimization

The primers were individually optimized using human reference DNA. Subsequently, all 21 primers were strategically pooled in two separate reactions based on predictions generated from the in silico PCR tool (Silica; Gear Genomics) [41] (https://www.gear-genomics.com/silica/). Following preliminary optimization using the human reference genome and pooled primer sets, the resulting amplicons were subjected to sequencing using ONT. Raw sequencing data were subsequently assessed using FastQC tool to evaluate the quality of the sequences. The results indicated that the quality scores across all bases and the quality score distribution over the sequences were favorable with a mean quality score above Q30 (> Q30) for read lengths greater than 4 kb (Figure S1). Moreover, comprehensive coverage of all target regions was confirmed covered, through visual inspection using the Integrative Genomics Viewer (IGV) tool [42] (Figure S2). To assess the precision or reproducibility of the system, sequencing of reference DNA was repeated. Variant profiling demonstrated complete concordance across runs, confirming 100% reproducibility and analytical precision.

### Variant prioritization

Following complete optimization of the panel using reference human DNA, the participant samples were screened using the developed system. Variants were prioritized and classified as VUS, and LP and pathogenic based on ACMG criteria. The results are listed in Table 2. Notably, the variants identified by the optimized ONT based system showed 100% concordance with those detected using Illumina-based sequencing, confirming 100% accuracy of the ONT platform in variant detection.

**Table 2:** Overview of clinical features, Follow-up, and genetic findings in patients with carnitine transport and cycle disorders

Patients	Clinical Features (diagnosis)	Treatment	Clinical features (Follow-up)	Variant detected by ONT	Variant detected by illumina	Heterozygosity	Pathogenicity	ACMG Criteria
1	RLE, encephalopathy and liver failure during infection, left ventricular dysfunction, hepatosteatosis intracranial bleeding, left hemiparesis, seizures	Dietary treatment MCT	Cardiomyopathy resolved, normal cardiac functions	CPT-1:NM_001876.4:c.1417 C > T:p.His473Tyr	CPT-1:NM_001876.4:c.1417 C > T:p.His473Tyr	Homozygous	LP	PM2, PP3
2	Recurrent pancreatitis, autism, psycho-motor retardation (moderate)	Dietary treatment with MCT	Type-I diabetes mellitus, speech problem, necessity for specialized education	CPT-1:NM_001876.4:c.1336G > T:p.Gly446Cys	CPT-1:NM_001876.4:c.1336G > T:p.Gly446Cys	Homozygous	VUS	PM2, PP3
3	RLE, encephalopathy, seizures	Dietary treatment with MCT	Speech problem necessitating special educational therapy until 6 years of age, speech difficulties resolved by the age of 6	CPT-1:NM_001876.4:c.1433 C > T:p.Ala478Val	CPT-1:NM_001876.4:c.1433 C > T:p.Ala478Val	Heterozygous	VUS	PM1, PM2, PP3
4	Elevated CK (4 months of age)	Dietary treatment with MCT	Rhabdomyolysis attack did not recur	CPT-1:NM_001876.4:c.740 C > T:p.Pro247Leu	CPT-1:NM_001876.4:c.740 C > T:p.Pro247Leu	Homozygous	LP	PM2, PP3
5	Encephalopathy during febrile illness, hepatosteatosis, ADHD	Dietary treatment with MCT	Speech problem, need for speech therapy until the age of 6	CPT-1:NM_001876.4:c.740 C > T:p.Pro247Leu	CPT-1:NM_001876.4:c.740 C > T:p.Pro247Leu	Homozygous	LP	PM2, PP3
6	Rhabdomyolysis, muscle weakness	L-carnitine treatment (20 mg/kg)	Recurrent rhabdomyolysis	CPT-2:NM_000098.3:c.338 C > T:p.Ser113Leu	CPT-2:NM_000098.3:c.338 C > T:p.Ser113Leu	Heterozygous	P	PM2, PP3, PP5
7	Rhabdomyolysis, muscle weakness, reduced exercise capacity	None	Recurrent rhabdomyolysis, not engaged in a course of treatment	CPT-2:NM_000098.3:c.338 C > T:p.Ser113Leu	CPT-2:NM_000098.3:c.338 C > T:p.Ser113Leu	Homozygous	P	PM2, PP3, PP5
8	Rhabdomyolysis muscle weakness, reduced exercise capacity	L-carnitine treatment (60 mg/kg).	Recurrent rhabdomyolysis (CK up to 96000 U/L), no adherance to the prescribed dietary regimen.	CPT-2:NM_000098.3:c.729_731del: p.Leu244del	CPT-2:NM_000098.3:c.729_731del: p.Leu244del	Heterozygous	VUS	PM1, PM2, PM4
				CPT-2:NM_000098.3:c.338 C > T:p.Ser113Leu	CPT-2:NM_000098.3:c.338 C > T:p.Ser113Leu	Heterozygous	P	PM2, PP3, PP5
9	Rhabdomyolysis, muscle weakness	None	Recurrent rhabdomyolysis, no adherance to the prescribed dietary regimen.	CPT-2:NM_000098.3:c.137 A > G:p.Gln46Arg	CPT-2:NM_000098.3:c.137 A > G:p.Gln46Arg	Homozygous	VUS	PM2, PP3
10	Rhabdomyolysis, muscle weakness, reduced exercise capacity, lipid storage myopathy	None	Recurrent rhabdomyolysis with exercise, hypothyroidism, renal calculi	CPT-2:NM_000098.3:c.338 C > T:p.Ser113Leu	CPT-2:NM_000098.3:c.338 C > T:p.Ser113Leu	Homozygous	P	PM2, PP3, PP5
11	Rhabdomyolysis, muscle weakness, reduced exercise capacity	None	Recurrent rhabdomyolysis, does not adhere to treatment	CPT-2:NM_000098.3:c.1261G > A:p.D421N	CPT-2:NM_000098.3:c.1261G > A:p.D421N	Heterozygous	VUS	PM2
12	Hypotonia, motor retardation	None	A heterozygous CPT-2 mutation and homozygous TRIP4 mutation (myopathy-related), symptoms may not be CPT-2 related. Tandem MS numeric results not available but reported as normal	CPT-2:NM_000098.3:c.1507 C > T:p.Arg503Cys	CPT-2:NM_000098.3:c.1507 C > T:p.Arg503Cys	Heterozygous	P	PM2, PM3, PM5, PP3
13	Rhabdomyolysis, muscle weakness	L-carnitine treatment (40 mg/kg)	Recurrent rhabdomyolysis, on triheptanoin treatment for 1 year (reduced metabolic attack frequency)	CPT-2:NM_000098.3:c.338 C > T:p.Ser113Leu	CPT-2:NM_000098.3:c.338 C > T:p.Ser113Leu	Homozygous	P	PM2, PP3, PP5
14	Hypoglycemia, encephalopathy during infection, hypertrophic CMP	L-carnitine treatment (4 g/day),	FC decreases without treatment, hypertrophic CMP improved with L-carnitine	SLC22A5:NM_003060.4:c.454G > C:p.Gly152Arg	SLC22A5:NM_003060.4:c.454G > C:p.Gly152Arg	Heterozygous	LP	PM2, PM5, PP2, PP3
				SLC22A5:NM_003060.4:c.506G > C:p.Arg169Pro	SLC22A5:NM_003060.4:c.506G > C:p.Arg169Pro	Heterozygous	P	PM1, PM2, PM5, PP2, PP3
15	Encephalopathy, seizures	L-carnitine treatment (4 g/day)	FC 0,5 $\:\mu\:$M//L at diagnosis, symptom free on L-carnitine treatment	SLC22A5:NM_003060.4:c.1519_1524del: p.Phe508_Leu509del	SLC22A5:NM_003060.4:c.1519_1524del: p.Phe508_Leu509del	Homozygous	VUS	PM2, PM4
				SLC22A5:NM_003060.4:c.1525 C > A:p.Leu509Ile	SLC22A5:NM_003060.4:c.1525 C > A:p.Leu509Ile	Homozygous	VUS	PM2, PP2
16	Asymptomatic diagnosis by expanded NBS	L-carnitine treatment (4 g/day)	FC 7 µmol/L in neonatal period, decreases after cessation of treatment, symptom free on L-carnitine treatment	SLC22A5:NM_003060.4:c.454G > C:p.Gly152Arg	SLC22A5:NM_003060.4:c.454G > C:p.Gly152Arg	Heterozygous	LP	PM2, PM5, PP2, PP3
17	Muscle weakness, dilated CMP	L-carnitine treatment (4 g/day)	Dilated CMP improved with treatment,	SLC22A5:NM_003060.4:c.1427T > G:p.Leu476Arg	SLC22A5:NM_003060.4:c.1427T > G:p.Leu476Arg	Homozygous	LP	PM1, PM2, PP2, PP3, PP5
18	Muscle weakness, reduced exercise capacity	L-carnitine treatment (4 g/day),	PSMR (Cerebellar hypoplasia), walks with support, using splints, muscle weakness did not recur on L-carnitine treatment	SLC22A5:NM_003060.4:c.249_250insACCGGCTCGCC: p.Tyr84Thrfs*50	SLC22A5:NM_003060.4:c.249_250insACCGGCTCGCC: p.Tyr84Thrfs*50	Homozygous	LP	PVS1, PM2
19	None, diagnosed by expanded NBS	L-carnitine treatment (3 g/day), FC 6 µmol/L, increases to 20 µmol/L with treatment	Symptom free on L-carnitine treatment.	SLC22A5:NM_003060.4:c.40T > C:p.Trp14Arg	SLC22A5:NM_003060.4:c.40T > C:p.Trp14Arg	Heterozygous	P	PS1, PM1, PM2, PP2, PP3
20	Dilated CMP	FC 6.18 µmol/L at diagnosis; decreases without treatment	Dilated CMP improved with treatment, symptom free on L-carnitine treatment	SLC22A5:NM_003060.4:c.217del: p.Asp73Thrfs*57	SLC22A5:NM_003060.4:c.217del: p.Asp73Thrfs*57	Heterozygous	LP	PVS1, PM2
				SLC22A5:NM_003060.4:c.254_265del: p.Arg85_Ile89delinsLeu	SLC22A5:NM_003060.4:c.254_265del: p.Arg85_Ile89delinsLeu	Heterozygous	VUS	PM1, PM2, PM4

The table summarizes the identified genetic variants, their zygosity, pathogenicity classification, and corresponding ACMG criteria used for classification. Notes: Variants listed are classified according to ACMG criteria (VUS, LP, and P). VUS: variant of unknown significance, LP: likely pathogenic, P: pathogenic, ONT: Oxford Nanopore Technologies; ACMG: American College of Medical Genetics and Genomics, RLE: Reye like episode, CK: creatine kinase, ADHD: Attention-Deficit/Hyperactivity Disorder, CMP: Cardiomyopathy, MCT: Medium-Chain Triglycerides, PSMR: Primitive Symmetric Motor Responses. FC: Free carnitine, none: no treatment offered

No variants were identified in the SLC25A20 gene within the cohort, consistent with prior Illumina-based sequencing results, which did not detect any SLC25A20 mutation in these patients. Of the 20 patients, 6 (30%) carried a single variant, 4 (20%), among which one of them was with two different homozygous variants, had compound heterozygous variants, and the remaining (50%) exhibited biallelic alterations, enabling a molecularly diagnosis of carnitine deficiency. Gene specific findings indicated that *CPT-1* was affected in 5 patients (25%), *CPT-2* in 8 patients (40%) and *SLC22A5* in 7 patients (35%).

The most frequently observed variants in Turkish patients included *CPT-1*:NM_001876.4: c.740 C > T:p.Pro247Leu (2/5;40%), *CPT-2*:NM_000098.3:c.338 C > T:p.Ser113Leu (5/8; 62.5%) and *SLC22A5*:NM_003060.4:c.454G > C:p.Gly152Arg (2/7; 28.6%). These findings underscore the utility of the developed panel for accurate molecular diagnosis of carnitine deficiency in this population.

The ONT-based CCD panel demonstrated high sequencing accuracy, successfully detecting all exonic and known pathogenic intronic variants across the cohort. A direct comparison with Illumina-based sequencing confirmed 100% concordance, validating the panel’s reliability and diagnostic accuracy. None of the patients in our cohort were identified as carriers of an SLC25A20 mutation based on previous Illumina-based sequencing results; therefore, no variants were detected for this gene. However, to ensure comprehensive panel design, primers targeting SLC25A20 were specifically developed and validated using human reference DNA. Although no individuals in this study were found to carry any variants in SLC25A20, the gene was incorporated into the CCD panel to enable its future application in broader diagnostic settings, ensuring its utility for patients who may present with CACT deficiency in prospective screenings. Performance evaluation of the ONT-based CCD panel demonstrated 100% sensitivity, 100% precision within the study cohort, underscoring the panel’s high analytical reliability and diagnostic accuracy.

### Comparative analysis of in silico prediction tools and clinical correlations

In addition to classifying variants based on standard criteria, a systematic comparison of in silico prediction tools, specifically MutPred2 and Missense3D, was utilized in order to assess the pathogenicity of VUS and LP variants. Table 3 summarizes the predictions for eight key variants across *CPT-1*, *CPT-2*, and *SLC22A5*. Overall, the predictions were concordant for 50% (4 out of 8) of the evaluated variants. For instance, for CPT-2:NM_000098.3:c.137 A > G:p.Gln46Arg and CPT-2:NM_000098.3:c.1261G > A:p.Asp421Asn, both tools indicated no major functional or structural disruption, suggesting a likely benign effect. However, clinical observations provide additional insights: the patient harboring the CPT-2:NM_000098.3:c.137 A > G:p.Gln46Arg variant presented with recurrent episodes of rhabdomyolysis and a characteristic acylcarnitine profile consistent with CPT2 deficiency, indicating potential pathogenicity despite the in silico benign prediction. Similarly, although SLC22A5:NM_003060.4:c.1525 C > A:p.Leu509Ile was predicted by both tools as not significantly affecting protein structure (MutPred2 score 0.48, Possibly Benign), the corresponding patient exhibited markedly reduced free carnitine levels (0.5 µmol/L), suggesting that this mutation may indeed contribute to the observed metabolic phenotype. In contrast, several CPT-1 variants demonstrated discrepancies between the tools. For example, CPT-1:NM_001876.4:c.1336G > T:p.Gly446Cys received a MutPred2 score of 0.70 (Possibly Damaging), whereas Missense3D indicated no major structural disruption. Similarly, CPT-1:NM_001876.4:c.1433 C > T:p.Ala478Val yielded a MutPred2 score of 0.65 (Possibly Damaging) with minimal structural change predicted by Missense3D.

**Table 3:** In Silico pathogenicity predictions for missense variants identified in the study cohort

Variant	Pathogenicity	Domain Localization	MutPred2 Prediction (Score/Category)	Missense3D Prediction (structural impact)	Interpretation Summary
CPT-1:NM_001876.4:c.1417 C > T:p.His473Tyr	LP	Cytoplasmic; topological domain (Figure S3)	Affected (0.72 / Possibly Damaging)	Not affected (Minimal structural change)	Likely Pathogenic; conflicting predictions underscore need for functional validation
CPT-1:NM_001876.4:c.1336G > T:p.Gly446Cys	VUS	Cytoplasmic; topological domain	Affected (0.70 / Possibly Damaging)	Not affected (No major disruption)	Conflicting predictions; additional in-vitro/clinical data are required to clarify significance
CPT-1:NM_001876.4:c.1433 C > T:p.Ala478Val	VUS	Cytoplasmic; topological domain	Affected (0.65 / Possibly Damaging)	Not affected (No major disruption)	Potentially damaging per MutPred2; structural modeling suggests minimal impact
CPT-1:NM_001876.4:c.740 C > T:p.Pro247Leu	LP	Cytoplasmic; topological domain	Affected (0.75 / Possibly Damaging)	Not affected (Minimal structural change)	Likely Pathogenic; clinical/biochemical findings support pathogenic role despite minor structural impact
CPT-2:NM_000098.3:c.137 A > G:p.Gln46Arg	VUS	Mitochondrial matrix; topological domain (Figure S4)	Not affected (0.45 / Likely Benign)	Not affected Not affected (No major disruption)	Uncertain significance; conflicting clinical presentation (rhabdomyolysis) suggests further functional study
CPT-2:NM_000098.3:c.1261G > A:p.Asp421Asn	VUS	Mitochondrial matrix; topological domain	Not affected (0.40 / Likely Benign)	Not affected (No major disruption)	Uncertain significance; additional correlation with biochemical data needed
SLC22A5:NM_003060.4:c.454G > C:p.Gly152Arg	LP	Transmembrane (Figure S5)	Affected (0.78 / Probably Damaging)	Affected (Significant structural rearrangement)	Likely Pathogenic; consistent damaging predictions, clinical improvement with L-carnitine supplementation
SLC22A5:NM_003060.4:c.1525 C > A:p.Leu509Ile	VUS	Transmembrane	Not affected (0.48 / Possibly Benign)	Not affected (No major disruption)	Uncertain significance; low free carnitine suggests pathogenic potential; additional functional data needed

The table presents computational analyses for the missense variants, including tools used for evaluation, predicted effects on protein structure and function, and pathogenicity classifications. Notes. Predictions were made using MutPred2 (functional impact scores) and Missense3D (structural alterations). MutPred2 Score/Category*: <0.5; likely benign, 0.5–0.75; possibly damaging and > 0.75; probably damaging. Missense3D Prediction: Identifies structural changes in proteins caused by amino acid substitutions; not affected: minimal or no predicted disruption in 3D conformation, affected: notable structural changes likely to impair function. VUS: variant of unknown significance, LP: likely pathogenic

Additionally, both CPT-1:NM_001876.4:c.1417 C > T:p.His473Tyr and CPT-1:NM_001876.4:c.740 C > T:p.Pro247Leu were flagged as potentially damaging by MutPred2 (scores of 0.72 and 0.75, respectively) but were considered to have only minimal structural impacts by Missense3D. Notably, the LP variant SLC22A5:NM_003060.4:c.454G > C:p.Gly152Arg was consistently predicted to be damaging (MutPred2 score 0.78, “Probably Damaging” with significant structural rearrangement per Missense3D). This variant’s pathogenicity is further supported by clinical data; the patient presented with hypertrophic cardiomyopathy that improved with L-carnitine supplementation and demonstrated decreased free carnitine levels in the absence of treatment.

For indel variants with unreported pathogenicity in the literature, we utilized SWISS-MODEL to predict the structural impact on the affected proteins. The analysis revealed significant alterations in protein structures, supporting the classification of these variants as pathogenic (Table 4). All corresponding web-based prediction outputs are provided in Figures S6-S13 and figures S14-S18, respectively.

**Table 4:**
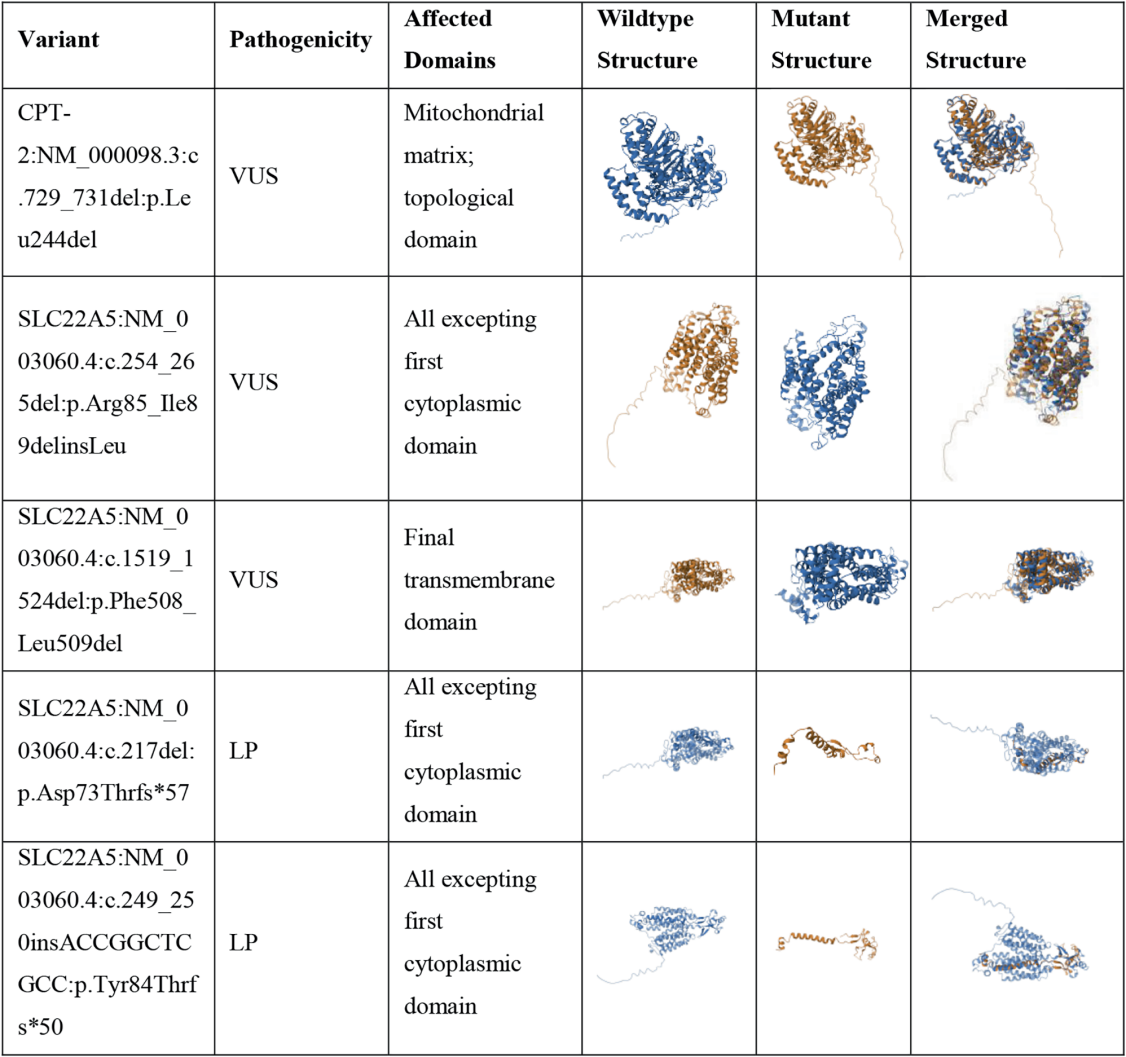
Structural alterations in proteins caused by indel variants identified in the study cohort

This table outlines the structural impacts of indel variants on proteins, including changes in 3D conformation, as assessed through computational modeling and structural alignment. Notes. Structural alterations were analyzed using *SWISS-MODEL* for mutant protein modeling and the Pairwise Structure Alignment tool from the Protein Data Bank (PDB) for comparison with wild-type structures. VUS: variant of unknown significance, LP: likely pathogenic

## Discussion

CCD are critical metabolic pathologies that may present and be diagnosed in the neonatal period often leading to life-threatening consequences if not diagnosed promptly. These disorders, particularly those involving fatty acid oxidation defects such as CCD are not currently part of the national newborn screening programs in Türkiye. However, pilot population-based screening programs have demonstrated their utility, suggesting a potential benefit for broader implementation in the future [42]. The diagnosis of CCD typically hinges on measuring specific metabolite levels as these provide critical biochemical evidence of impaired fatty acid metabolism. However, due to the complex nature of CCD, particularly those involving systemic carnitine deficiency; SCD, a differential approach is essential, where molecular diagnostic tools become increasingly important [43]. NGS has emerged as a powerful tool for the molecular diagnosis of the CCD facilitating the identification of both known and novel genetic mutations [44]. So-called second-generation systems including Illumina (Illumina Inc., USA), MGI (MGI, China) and Thermo Scientific (Thermo Scientific, USA) platforms are widely used in the clinics. By these platforms, targeted gene sequencing as gene panels, clinical exome sequencing (CES) or whole exome sequencing (WES) could be performed [45]. Nevertheless, such systems necessitate costly facilities, and are time consuming and laborious. Moreover, they are able to sequence the target regions as short fragments, which may cause to skip the structural variants [46]. Thus, there is a growing need for alternative sequencing approaches that can improve diagnostic yield, efficiency and cost-effectiveness. In the current study, we developed an amplification-based sequencing panel adaptable to the ONT platform for screening CCD genes. ONT, as a third-generation sequencing system, has several advantages over second-generation systems, including its long-read capability, relatively low cost, and affordability of the devices [47]. The cost-effectiveness of the ONT based gene panels has been reported in terms of both time and cost in the literature [48]. Hence, the low initial investment cost, multiplexing samples, and shorter time requirements are the benefits of the CCD panel described in our work.

To design the system called CCD panel, we focused on four key genes, *CPT-1*, *CPT-2*, *SLC22A5* and *SLC25A20*, recognized for their roles in carnitine transport and fatty acid oxidation. This amplification-based sequencing panel was carefully designed to be compatible with the ONT platform, enabling a comprehensive and efficient approach to identifying genetic variants associated with CCD. By focusing on these key genes, the CCD panel aims to enhance diagnostic capabilities and offers a more feasible method for detecting mutations within these critical pathways. To ensure comprehensive variant detection, we designed primers for long range PCR which amplicon sizes ranging from 4 to 7 kb, ensuring that they were capable of amplifying not only the exons but also exon-intron boundaries, UTRs and intronic regions that may harbor pathogenic or LP variants as identified in the ClinVar database. Following initial monoplex trials, the primers were pooled in the two tubes and multiplex PCR was optimized using human reference DNA in conjunction with ONT sequencing. Quality assessment (Figure S1), revealed that while the quality scores were lower when compared to Illumina data (www.bioinformatics.babraham.ac.uk) such diminished read-based quality scores are characteristic of the ONT platform, as reported previously [22, 49]. As expected, the overall read lengths ranged between 4 and 7 kb (Figure S1). It is important to note that while the per-base quality scores of ONT data are generally lower, the long-read capability of ONT technology compensates by enabling comprehensive target coverage and the resolution of complex genomic regions. In our study, despite lower raw quality scores, we achieved complete coverage of target regions and 100% concordance in variant detection relative to Illumina-based results, confirming its accuracy and reliability, hence demonstrating that the lower per-base quality does not compromise diagnostic accuracy. Moreover, recent advancements in base-calling algorithms (e.g., Guppy and Bonito) and ongoing improvements in library preparation protocols are expected to further enhance data quality, thereby reinforcing the clinical utility of ONT-based diagnostic approaches. Despite these successes, the read depths exhibited significant variation between amplicons (Figure S2). In an attempt to address this issue, we further optimized the primer concentrations; however, these efforts were not able to overcome the issue; likely due to the inherent limitations of the multiplex long-range PCR [50]. Nevertheless, the read depth variability did not compromise our ability to screen patient samples, and the assay performance remained robust.

We validated the CCD panel by screening 20 patients with previously confirmed molecular diagnoses obtained through Illumina-based sequencing. A 100% concordance between the two platforms underscored the reliability and diagnostic utility of the CCD multi-gene diagnostic kit. Notably, heterozygosity in the ONT system was defined by a minor allele frequency (MAF) threshold above 0.60, highlighting the necessity of clinical validation for in-house designed ONT panels through comparison with second-generation NGS platforms. Although the validation results were promising, further testing in a larger cohort is required to robustly establish the panel’s clinical applicability.

In terms of variant evaluation, while SLC22A5, CPT-1 and CPT-2 variants were successfully detected, no pathogenic variants were found in SLC25A20 within the study cohort. As we had no patients with confirmed SLC25A20 mutations, the primers were validated using human reference DNA to ensure their performance for future diagnostic applications. All patients presented at least one variant with unknown significance in *CPT-1*, *CPT-2* or *SLC22A5* genes. Specifically, six patients carried only one heterozygous variant (Table 2), which may not explain the genetic basis of the autosomal recessive CCD. Importantly, the possible second variant was also not detected by Ilumina platform. Although this status could be a result of any structural or intronic non-reported variants that could not be detected by both ONT and Illumina platforms, single heterozygous variants in *CPT-2* and *SLC22A5* were associated with CCD [51]. Of particular interest, three patients with a homozygous *CPT-2*:NM_000098.3:c.338 C > T:p.Ser113Leu variant received an already known molecular diagnosis, a variant frequently reported in the literature [52, 53]. Similarly, *SLC22A5*:NM_003060.4:c.454G > C:p.Gly152Arg variant, also reported in Turkish patients was predicted to be pathogenic in this study [54] (Table 3). Additionally, the LP variant according to the ACMG criteria [32], CPT-1:NM_001876.4:c.740 C > T:p.Pro247Leu was predominantly detected in our cohort although it has not been reported in the literature or databases. This variant was predicted to impact the protein structure/function according to MutPred2, though Missense3D did not support this prediction (Fig. 3).

Our findings highlight the diagnostic value of ONT-based sequencing for CCDs. The panel identified several recurrent mutations in our study cohort. Furthermore, we analyzed the potential pathogenicity of missense and indel variants that were not previously reported or confirmed as pathogenic, using in silico prediction tools. For the missense variants, predictions from MutPred2 and Missense3D tools showed significant differences. The Missense3D tool [36] which required a 3D structure of the proteins was limited by the lack of experimental models. To overcome this, we utilized 3D structures generated by AlphaFold [37], though this approach may have contributed to the higher frequency of non-affected proteins predicted by Missense3D tool. For instance, the His473 residue in CPT-1 protein, which is located in the catalytic site of the protein [55], suggesting that alterations close to this site could be pathogenic. Based on this, variants such as *CPT-1*:NM_001876.4: c.1336G > T:p.Gly446Cys and *CPT-1*:NM_001876.4: c.1433 C > T:p.Ala478Val were predicted by MutPred2 to potentially affect protein function (Table 2). Conversely, variants like *CPT2*:NM_000098.3:c.137 A > G:p.Gln46Arg, *CPT2*:NM_000098.3:c.1261G > A:p.Asp421Asn and *SLC22A5*:NM_003060.4:c.1525 C > A:p.Leu509Ile were not predicted to be pathogenic by MutPred2 tool, requiring further molecular validation to confirm the pathogenicity. For example, CPT2:NM_000098.3:c.137 A > G:p.Gln46Arg was predicted to be benign in silico, but the patient exhibited recurrent rhabdomyolysis, suggesting a possible disease association, and SLC22A5:NM_003060.4:c.1525 C > A:p.Leu509Ile had no predicted structural impact, yet the patient had markedly reduced free carnitine levels (0.5 µmol/L). Regarding indel variants, we modeled the affected proteins and compared them with wild-type counterparts. These indels were found to cause early stop codons, leading to significant structural disruptions, which could be considered as loss-of-function mutations (Table 4). Overall, these findings emphasize the importance of using multiple in silico tools to assess the pathogenicity of uncharacterized variants and while also highlighting the need for experimental validation to confirm their roles in disease. Although, in silico predictions provide valuable insights, the observed 50% concordance among tools illustrates the limitations of relying solely on computational methods. Integrating multiple prediction approaches with clinical and biochemical data, exemplified by consistent finding for the SLC22A5 p.Gly152Arg variant, yields a more robust and reliable framework for variant interpretation.

The CCD multi-gene diagnostic kit represents a scalable and accessible diagnostic approach for carnitine transport and cycle disorders, particularly valuable in regions with limited resources and high genetic diversity. Its compatibility with ONT technology offers a promising alternative to costly second-generation sequencing platforms, providing unique advantages such as the ability to resolve complex genomic regions and detect structural variants that are often missed by short-read methods. Our panel demonstrated robust detection of clinically relevant variants in CPT-1, CPT-2, and SLC22A5. While, the CCD panel provides comprehensive coverage of clinically relevant regions, it does not include complete intronic sequences. This study highlights the efficiency and cost-effectiveness of ONT-based targeted sequencing for CCD diagnostics. The estimated cost per patient, based on a cohort of 96 patients (with consumables scaled accordingly) was approximately 35 USD for the four- gene panel. The total processing time, excluding sequencing, was approximately 6 h. When compared to other ONT-based gene panels [56], this approach demonstrated lower costs, and both turnaround time and expenditure were substantially reduced relative to second- generation NGS platforms [57]. This targeted sequencing strategy optimizes diagnostic sensitivity and cost-efficiency, making it a practical and scalable option for routine clinical use. Future iterations may incorporate hybrid capture techniques or WGS-based strategies to expand genomic coverage where clinically warranted.

To fully exploit the benefits of ONT’s long-read capability, future experimental efforts will focus on applying our panel to samples known to harbour complex variants, thereby further refining diagnostic accuracy and clinical applicability. Additionally, future directions should include functional validation using protein expression studies or cellular models to confirm the pathogenicity of novel variants, as well as panel expansion to encompass related metabolic pathways for improved diagnostic yield. Ultimately, implementing this kit in clinical practice could reduce the diagnostic odyssey, enable earlier interventions, and mitigate long-term complications associated with delayed diagnoses.

### Study limitations and future directions

Despite the inherent amplification bias associated with the long-range PCR, which can result in variable read depth across different amplicons, our assay achieved complete coverage of all target regions and reliably detected clinically relevant variants. While this variability did not compromise the overall diagnostic accuracy of our panel, it may affect variant detection sensitivity, particularly for low-abundance alleles. To improve sequencing uniformity and reduce potential biases, further refinement of primer design or adoption of alternative enrichment strategies, such as hybrid-capture based methods, may be warranted.

While the ONT platform offers advantages in long-read sequencing and real-time data acquisition, it presents certain limitations. One notable constraint is amplicon length: excessively long amplicons can result in a phenomenon known as the suspension bridge effect, in which sequencing depth is disproportionately higher at the end of the amplicons, while coverage decreases in the central regions [22]. Our findings suggest that suspension bridge effect limits the feasibility of amplifying entire genes as single amplicons when gene length exceeds approximately 20 kbp. To ensure even coverage across all target regions, a multiplex long-range PCR strategy is therefore required. While the SLC25A20 gene was incorporated into the panel design, no pathogenic variants were detected in our patient cohort. The panel performance for SLC25A20 was therefore validated using human reference DNA. Future studies involving patients with confirmed SLC25A20 mutations are needed to fully evaluate its clinical utility and diagnostic accuracy. While, our panel effectively captured all regions with known pathogenicity, it did not include all intronic sequences, which may harbor regulatory or splicing-disrupting variants potentially relevant to disease pathogenesis.

## Conclusion

This study demonstrates the reliability and diagnostic utility of third-generation sequencing technology, specifically ONT, for the detection of CCDs, while acknowledging the platform’s inherent technical limitations and amplification-related biases. The ONT-adapted CCD multi-gene diagnostic kit, showed complete concordance with Illumina-based sequencing and effectively detected a broader spectrum of genetic variants, including novel mutations in *CPT-1*,* CPT-2*, and *SLC22A5* genes. These findings underscore the advantage of ONT in providing comprehensive genetic insights, which are critical for a more accurate diagnosis, especially in complex disorders where other platforms may miss structural or intronic variations. By integrating ONT-based targeted sequencing into routine molecular diagnostics, second-tier newborn screening, and targeted rare disease testing, this approach offers a cost-effective, scalable, and rapid diagnostic solution. Importantly, SLC25A20 was incorporated into the panel to ensure adaptability for future clinical use. These findings reinforce the importance of ONT sequencing as a first-line diagnostic tool, particularly in resource-limited settings where early and accurate genetic diagnosis can significantly improve patient outcomes and reduce the diagnostic odyssey for individuals with CCDs.

## Electronic supplementary material

Below is the link to the electronic supplementary material.

Supplementary Material 1

## Data Availability

The research team gathered the data described in this manuscript during the study period. Due to the nature of the research and legal restrictions, the supporting data collected is not publicly available but can be obtained from the corresponding authors upon reasonable request.

## Abbreviations

PCD: Primary Carnitine Deficiency
SCD: Secondary Carnitine Deficiency
CACT: Carnitine-Acylcarnitine Translocase
CCD: Carnitine Transport and Cycle Disorders
MS/MS: Tandem Mass Spectrometry
NGS: Next-Generation Sequencing
ONT: Oxford Nanopore Technology
BTD: Biotinidase Deficiency
EDTA: Ethylenediaminetetraacetic Acid
PCR: Polymerase Chain Reaction
VCF: Variant Call Format
ACMG: American College of Medical Genetics
VUS: Variants Of Unknown Significance
LP: Likely Pathogenic
PDB: Protein Data Bank
IGV: Integrative Genomics Viewer
CES: Clinical Exome Sequencing
WES: Whole Exome Sequencing
MAF: Minor Allele Frequency
IGV: Integrative Genomics Viewer
WGS: Whole Genome Sequence
