# Supplementary material for: Development and validation of a carnitine cycle and transport disorders (CCD) panel: an ONT-compatible multi-gene diagnostic kit for newborn and selective screening
Source: Orphanet J Rare Dis. 2025 May 26;20:250. doi: 10.1186/s13023-025-03775-4 (PMC12105206; doi:10.1186/s13023-025-03775-4)
Supplement: Supplementary file 1 — Supplementary Material 1 [file 13023_2025_3775_MOESM1_ESM.docx]

**Development and Validation of a Carnitine Cycle and Transport Disorders (CCD) Panel: An ONT-Compatible Multi-Gene Diagnostic Kit for Newborn and Selective Screening**

Gökçe Akan^1^, Mehmet Cihan Balcı^2^, Gülten Tuncel^1^, Meryem Karaca^2^, Hasan Hüseyin Kazan^3^, Ahmet Çağlar Özketen^1^, Özge Özgen^4^, Gülden Fatma Gökçay^2,4,5^ & Fatmahan Atalar^4,5,*^

^1^DESAM Institute, Near East University, Mersin 10, Türkiye

^2^Division of Pediatric Nutrition and Metabolism, Istanbul Medical Faculty, Istanbul University, Istanbul, Türkiye

^3^Department of Medical Biology, Gulhane Faculty of Medicine, University of Health Sciences, Ankara, Türkiye

^4^Rare Diseases Research Laboratory, Istanbul Medical Faculty, Istanbul University, Istanbul, Türkiye

^5^Department of Rare Diseases, Child Health Institute, Istanbul University, Istanbul, Türkiye


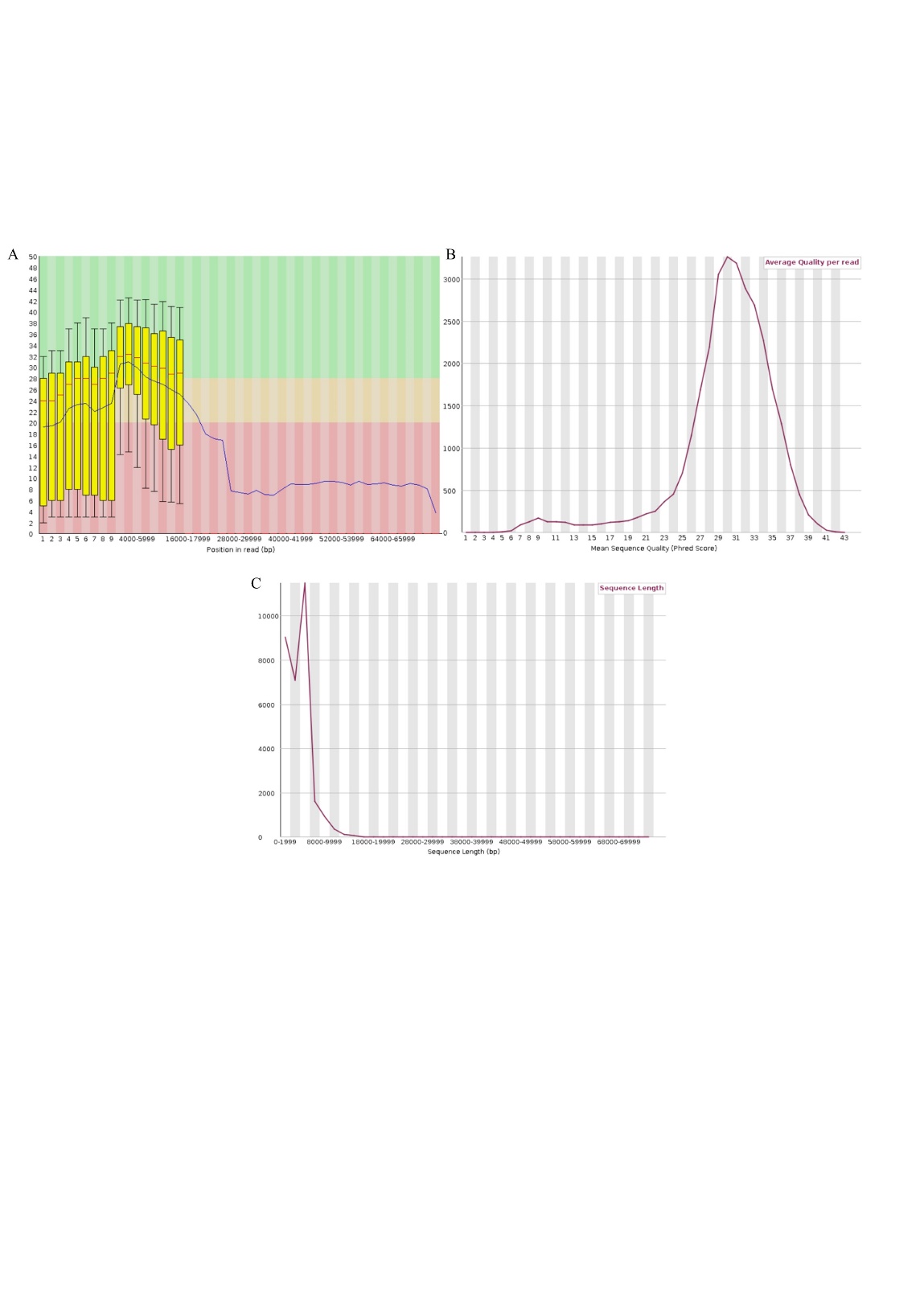


**Figure S1.** Quality parameters of the raw data. A. Quality scores across all bases. B. Quality score distribution over all sequences. C. Distribution of sequence lengths over all sequences.


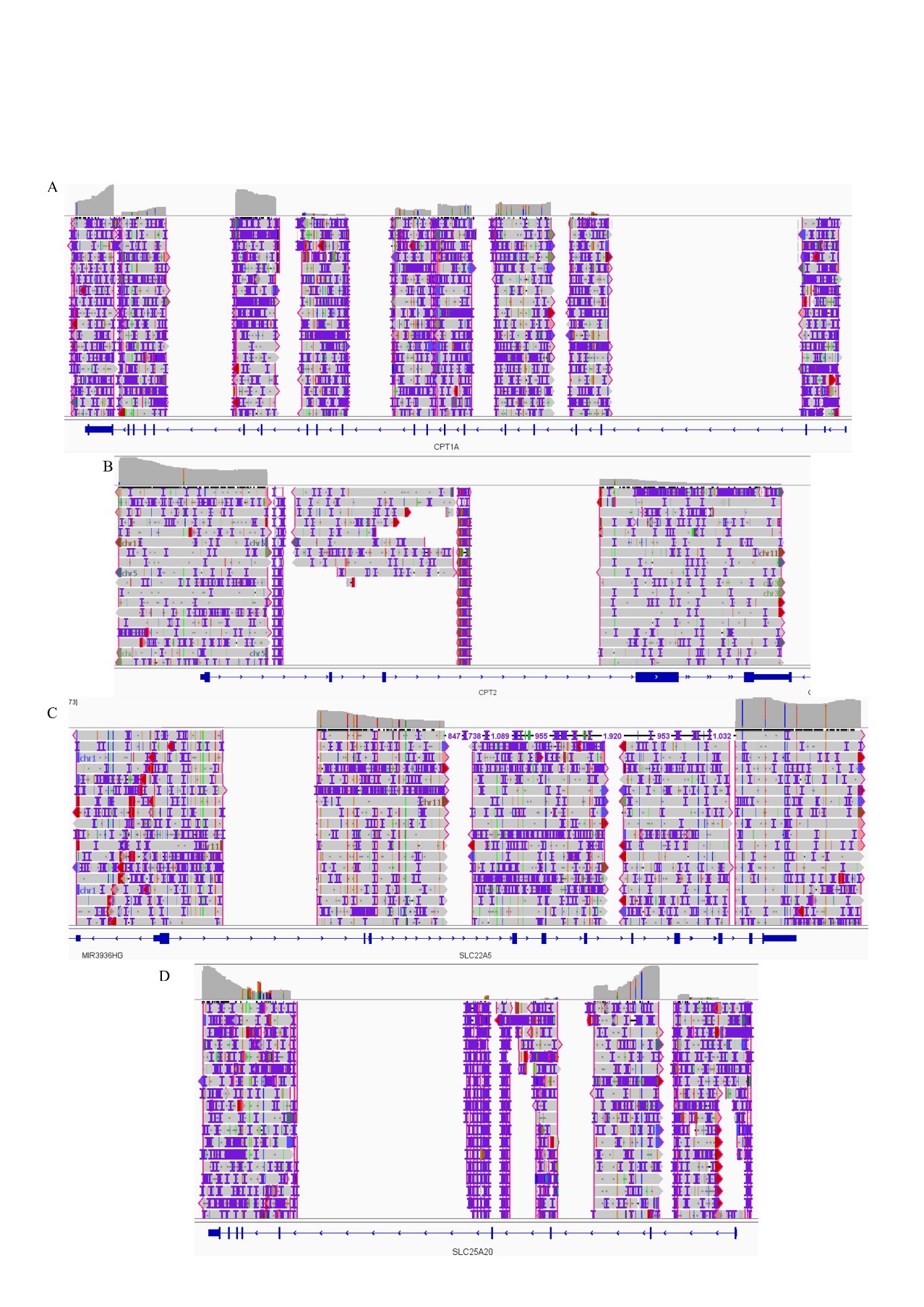


**Figure S2.** Coverages of the target regions illustrated by IGV tool. A. *CPT1A*, B. *CPT2*, C. *SLC22A5* and D. *SLC25A20*.


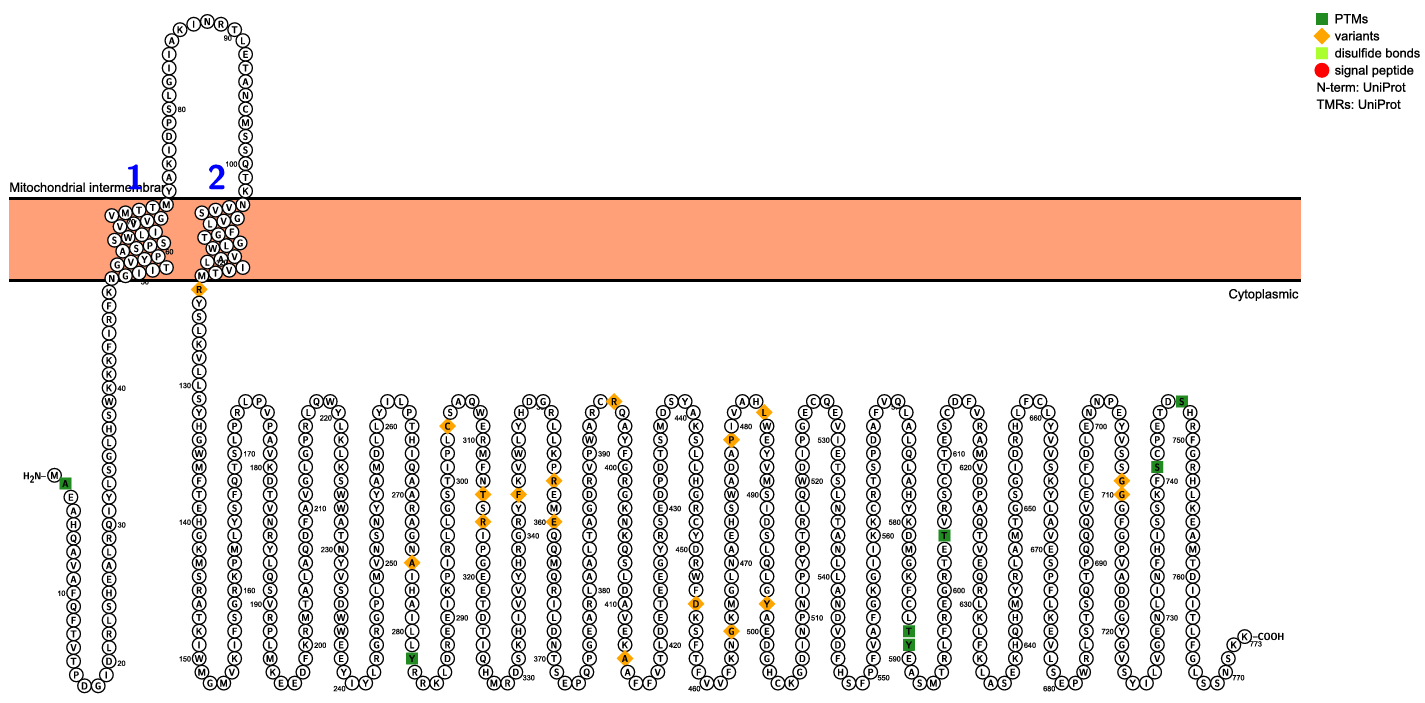


**Figure S3.** Structure and functional localization of the domains of the CPT1A protein.


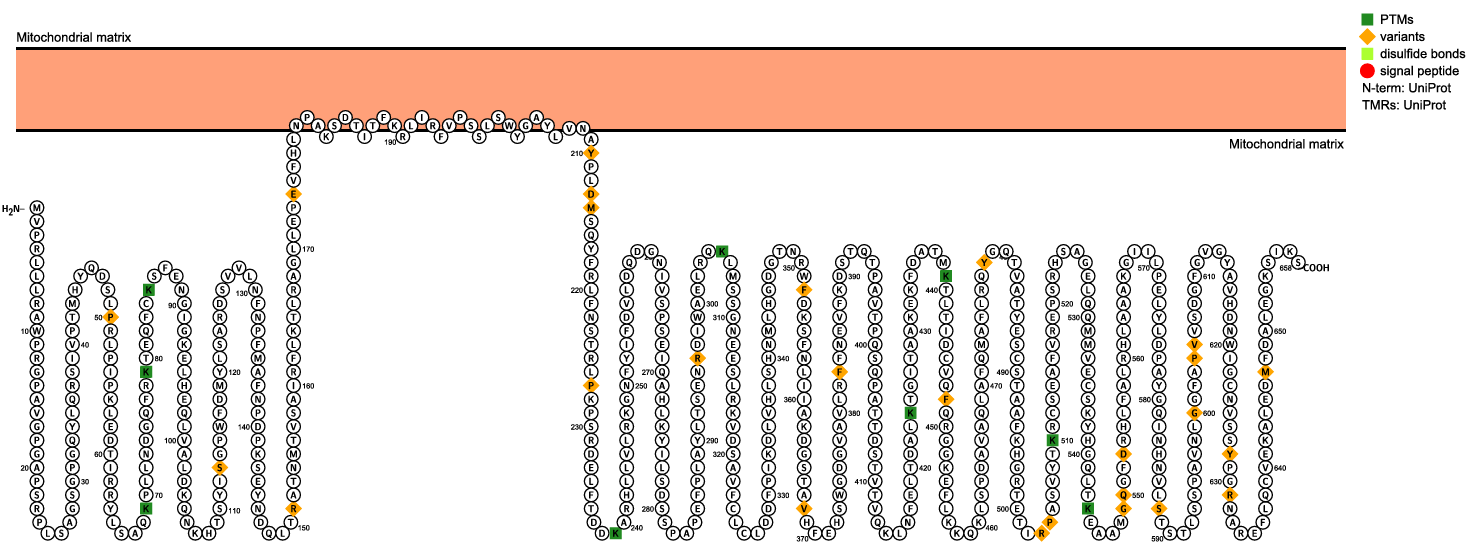


**Figure S4.** Structure and functional localization of the domains of the CPT2 protein.


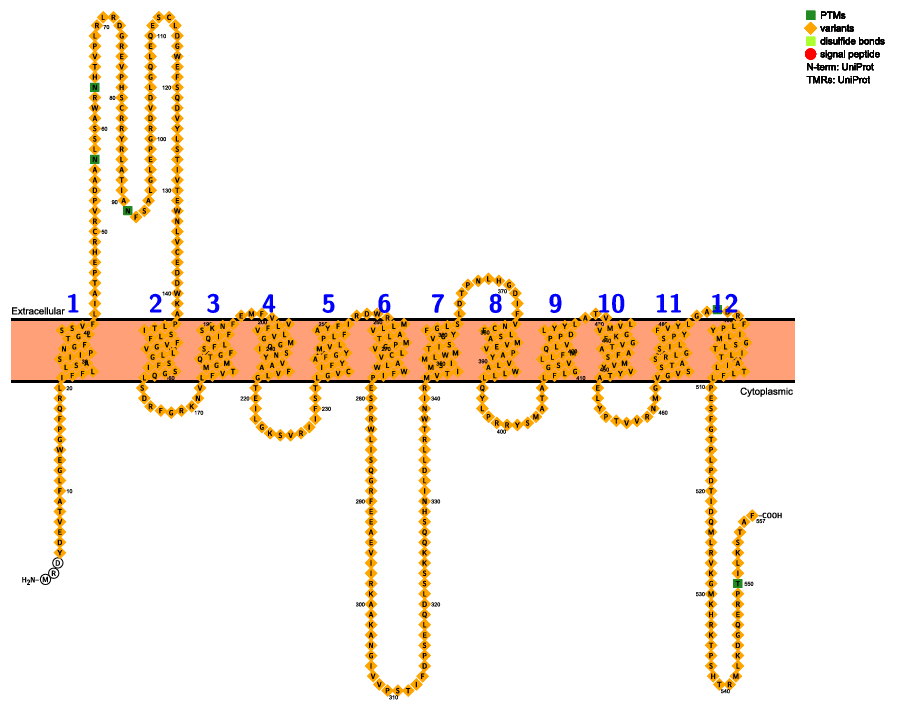


**Figure S5.** Structure and functional localization of the domains of the SLC22A5 protein.


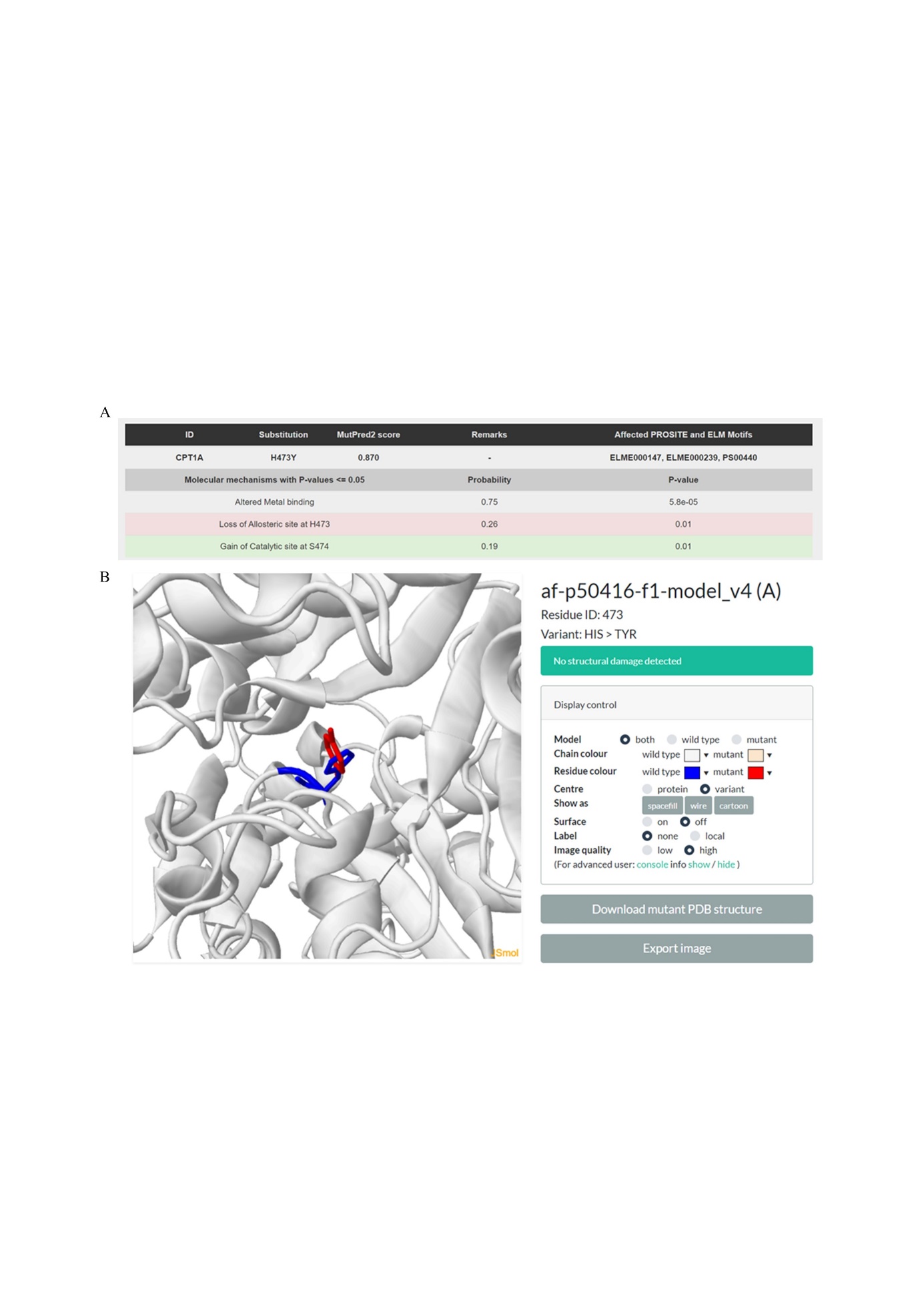


**Figure S6.** Prediction of pathogenicity of CPT1A:NM_001876.4:c.1417C>T:p.His473Tyr variant. A. by MutPred2 and B. by Missense3D.


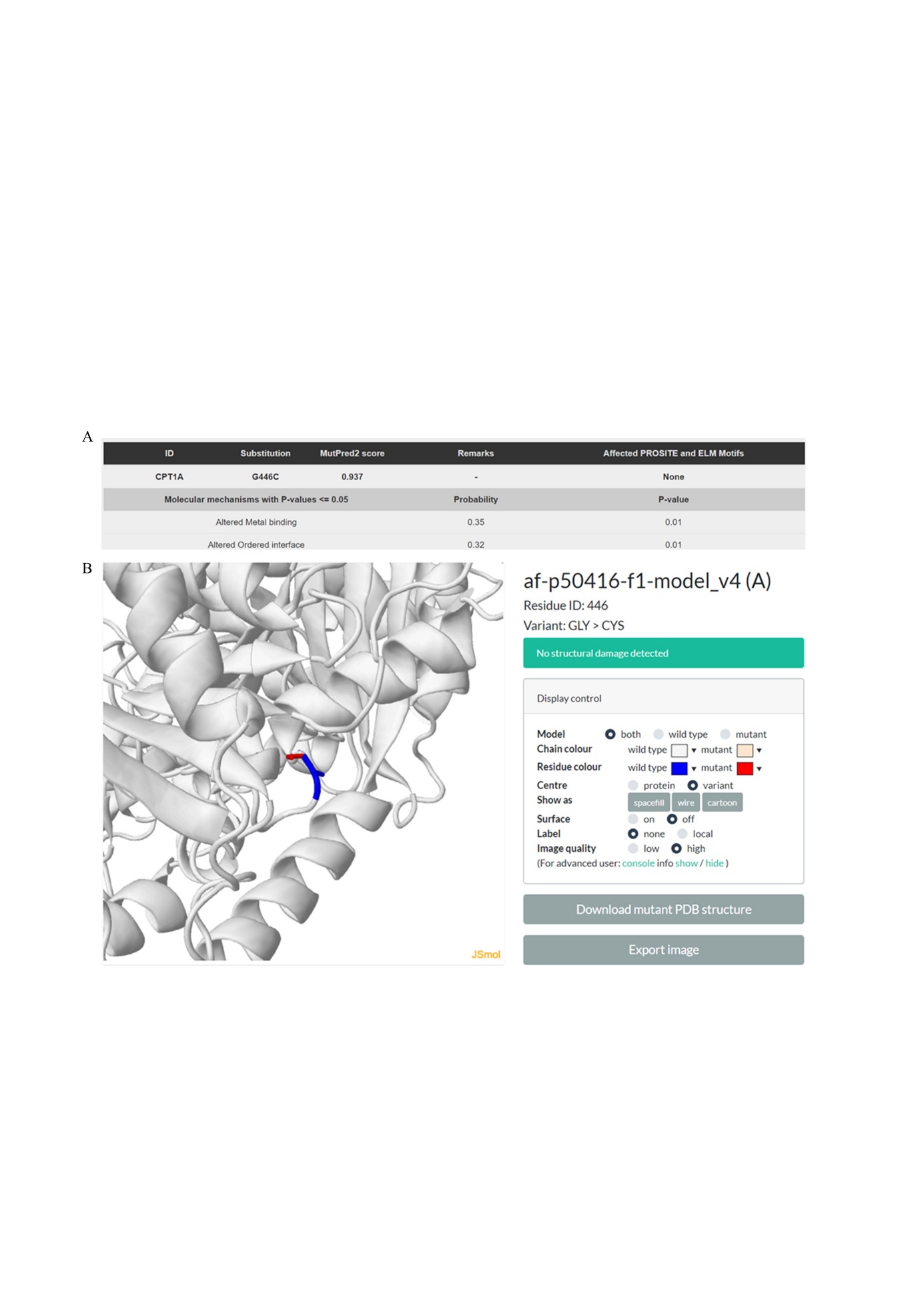


**Figure S7.** Prediction of pathogenicity of CPT1A:NM_001876.4:c.1336G>T:p.Gly446Cys variant. A. by MutPred2 and B. by Missense3D.


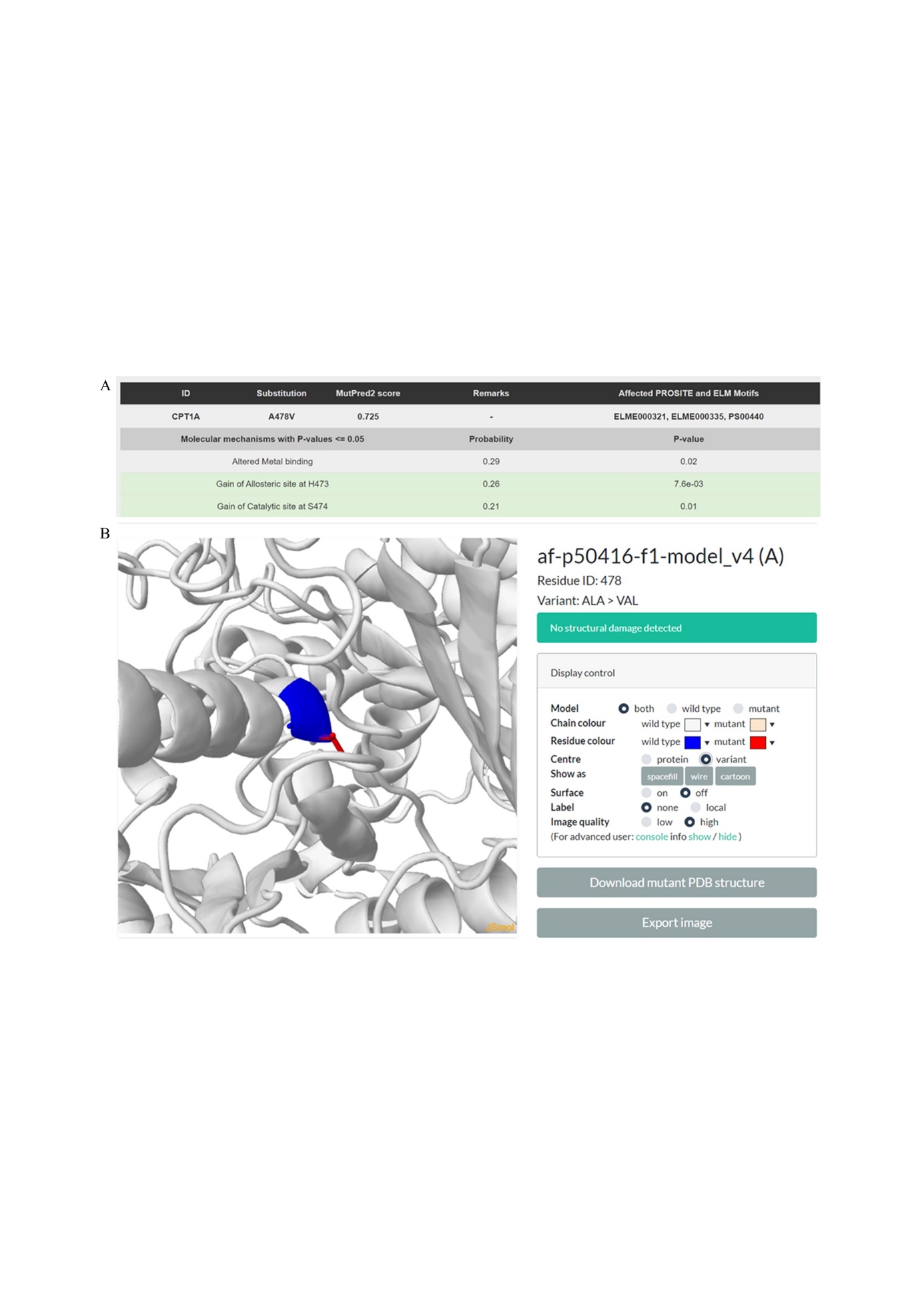


**Figure S8.** Prediction of pathogenicity of CPT1A:NM_001876.4:c.1433C>T:p.Ala478Val variant. A. by MutPred2 and B. by Missense3D.


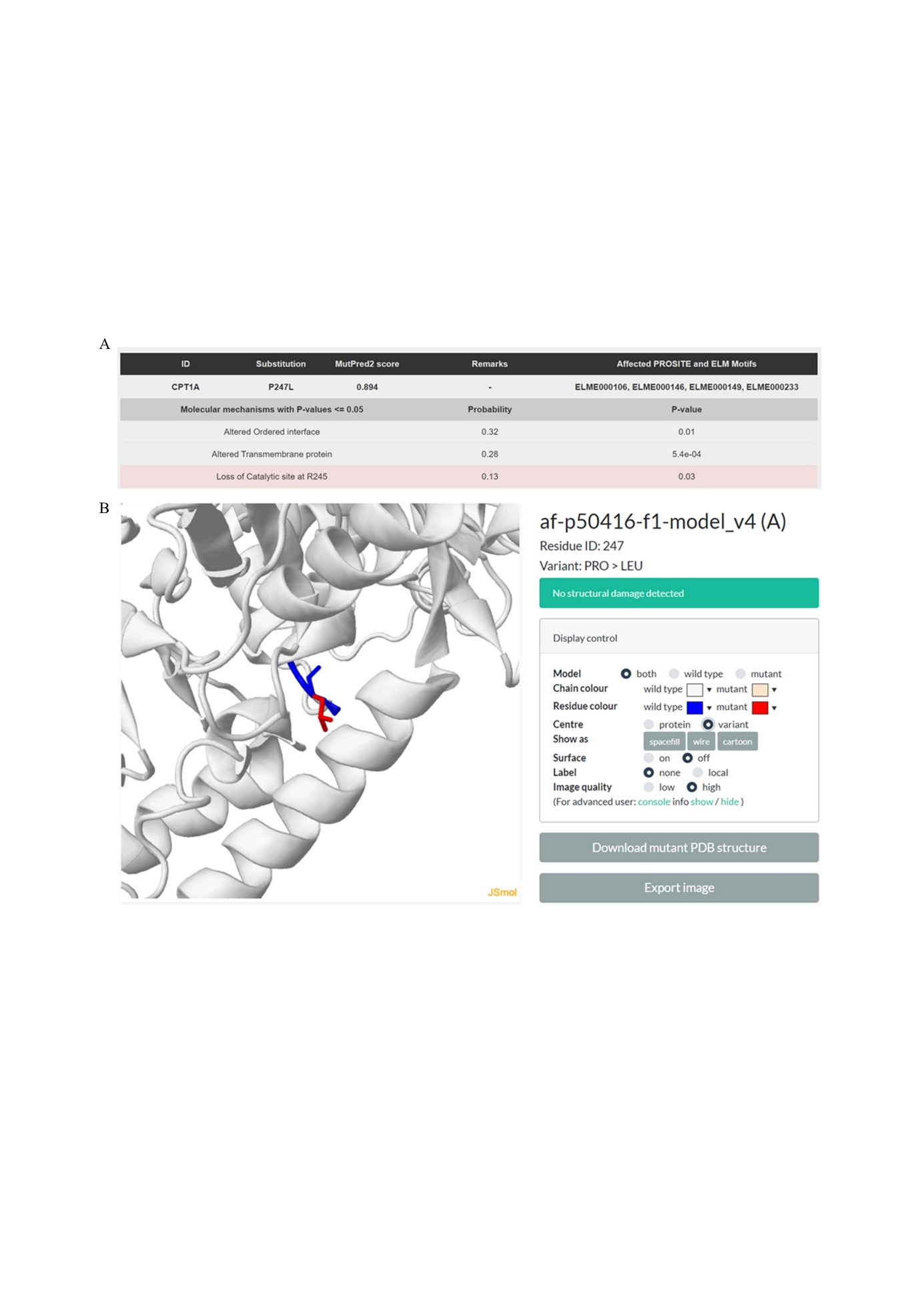


**Figure S9.** Prediction of pathogenicity of CPT1A:NM_001876.4:c.740C>T:p.Pro247Leu variant. A. by MutPred2 and B. by Missense3D.


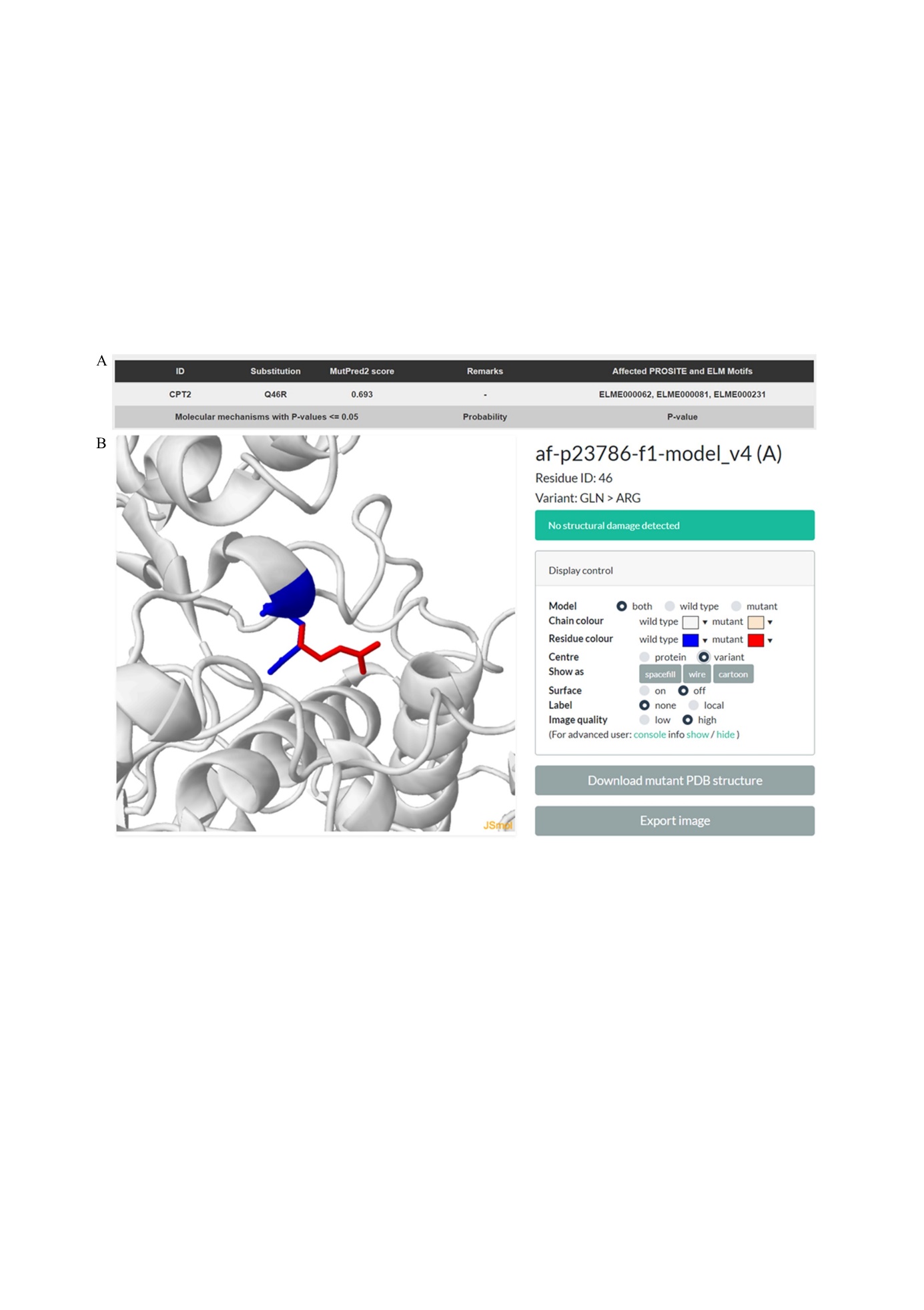


**Figure S10.** Prediction of pathogenicity of CPT2:NM_000098.3:c.137A>G:p.Gln46Arg variant. A. by MutPred2 and B. by Missense3D.


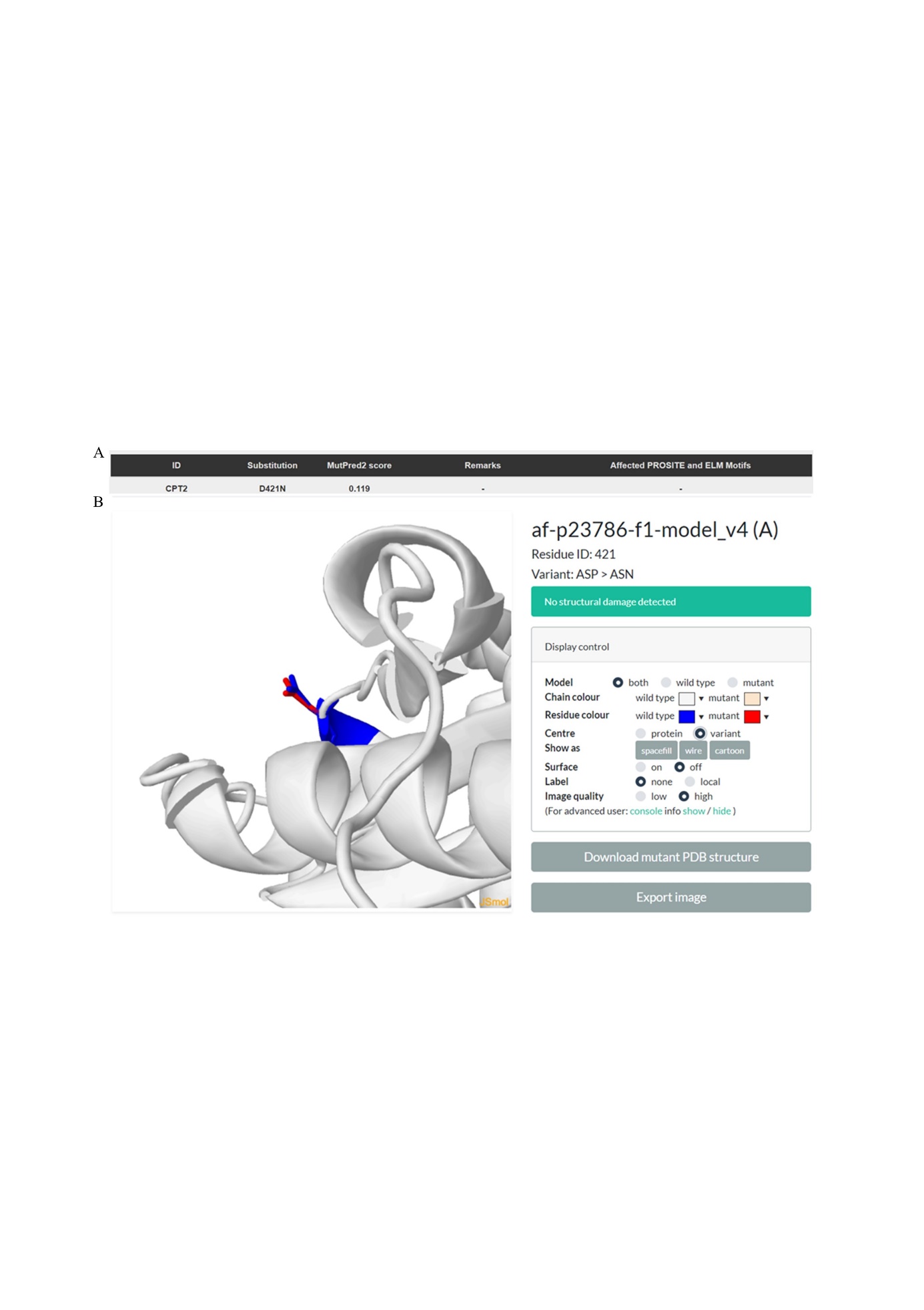


**Figure S11.** Prediction of pathogenicity of CPT2:NM_000098.3:c.1261G>A:p.D421N variant. A. by MutPred2 and B. by Missense3D.


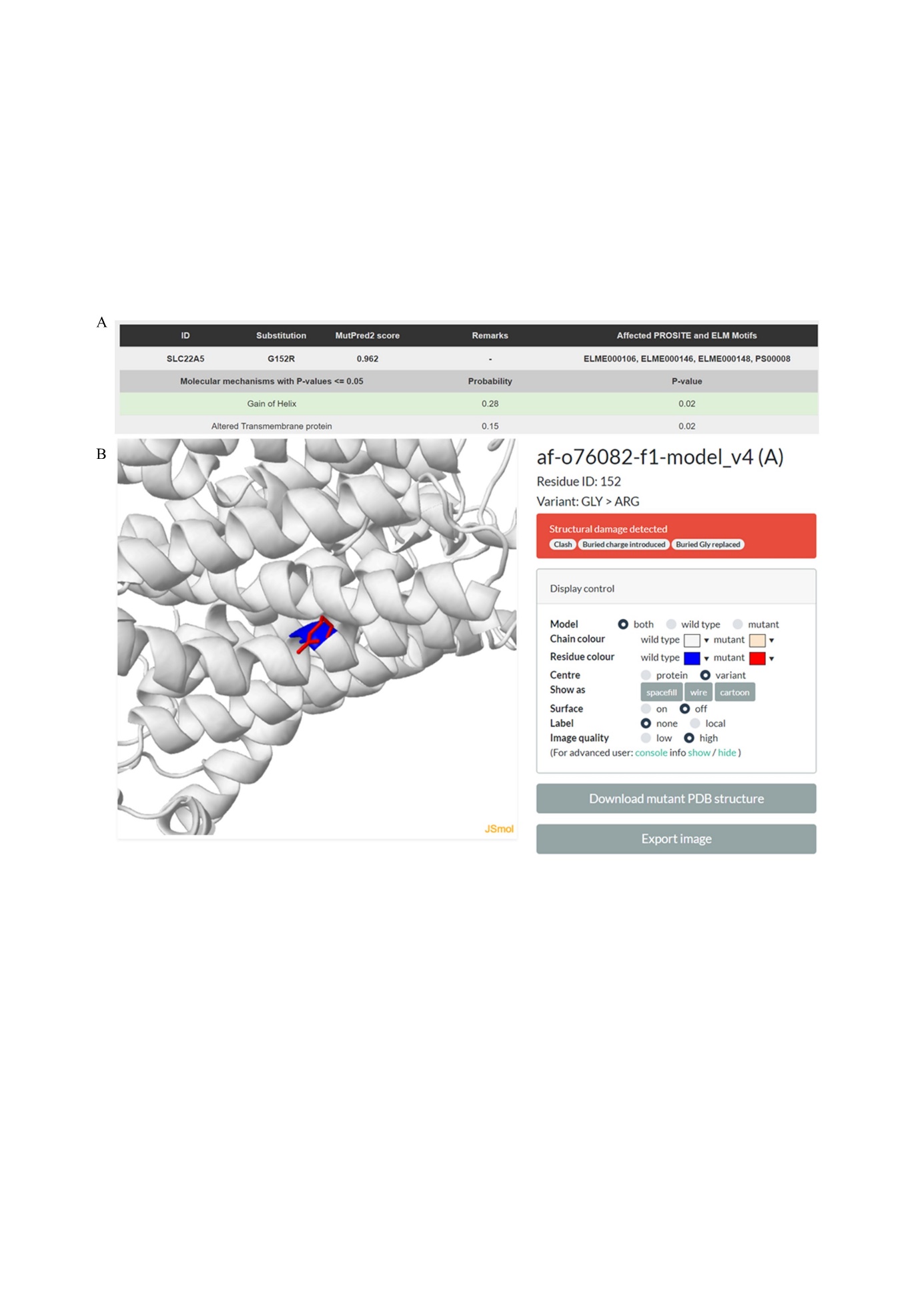


**Figure S12.** Prediction of pathogenicity of SLC22A5:NM_003060.4:c.454G>C:p.Gly152Arg variant. A. by MutPred2 and B. by Missense3D.


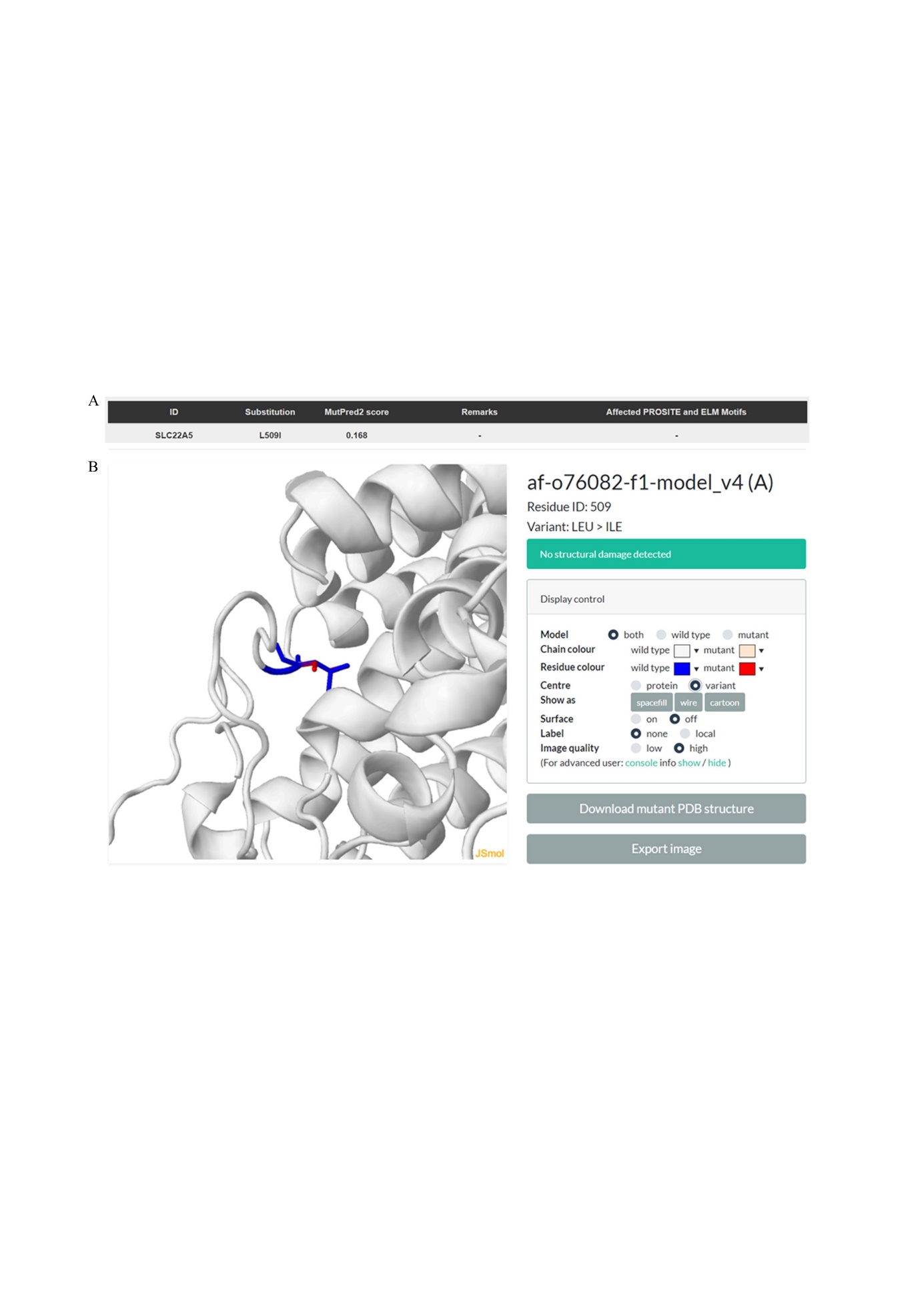


**Figure S13.** Prediction of pathogenicity of SLC22A5:NM_003060.4:c.1525C>A:p.Leu509Ile variant. A. by MutPred2 and B. by Missense3D.


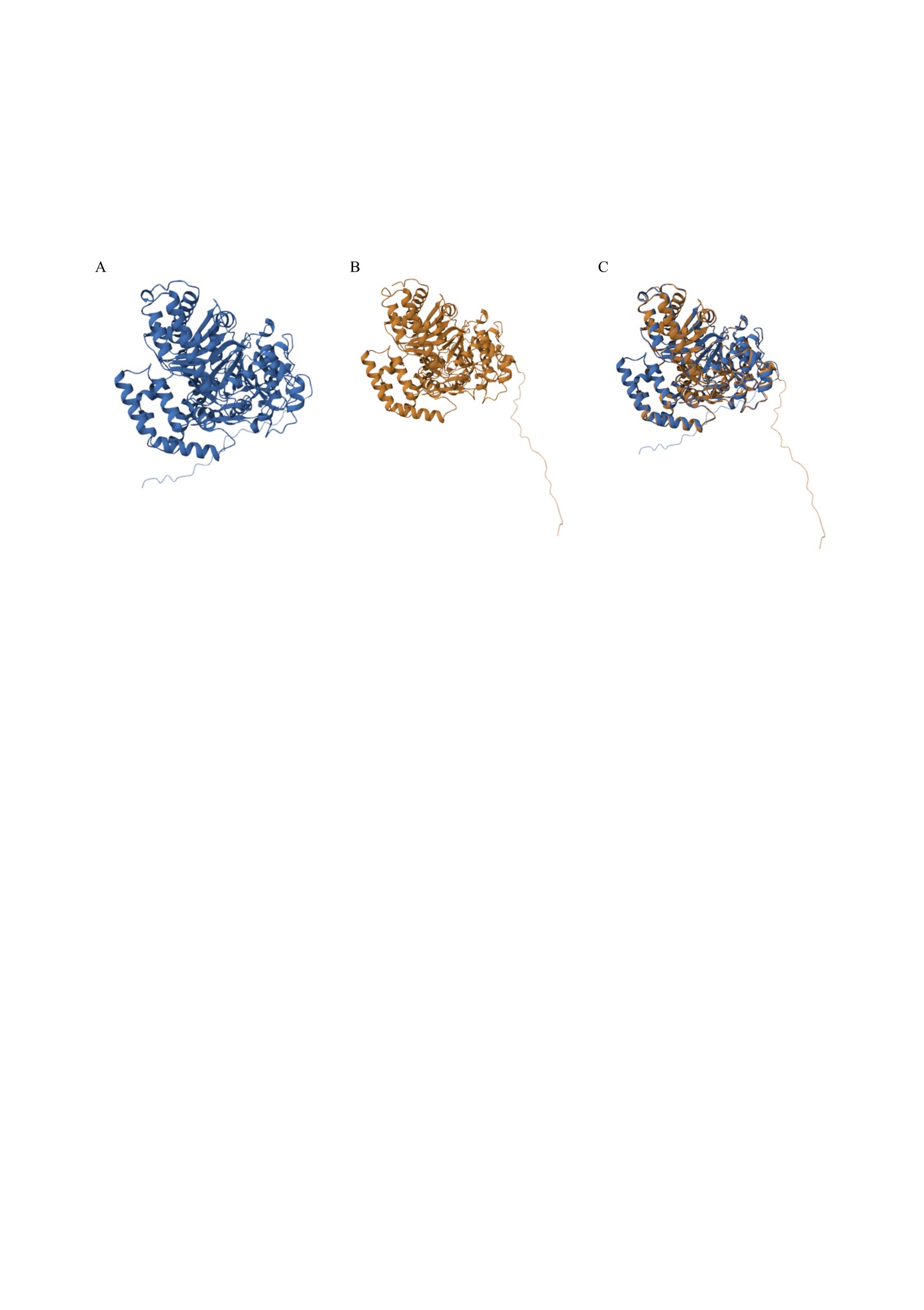


**Figure S14.** Effect of CPT2:NM_000098.3:c.729_731del:p.Leu244del variant on 3D structure of the CPT2 protein. A. Wildtype, B. Mutant and C. Merge.


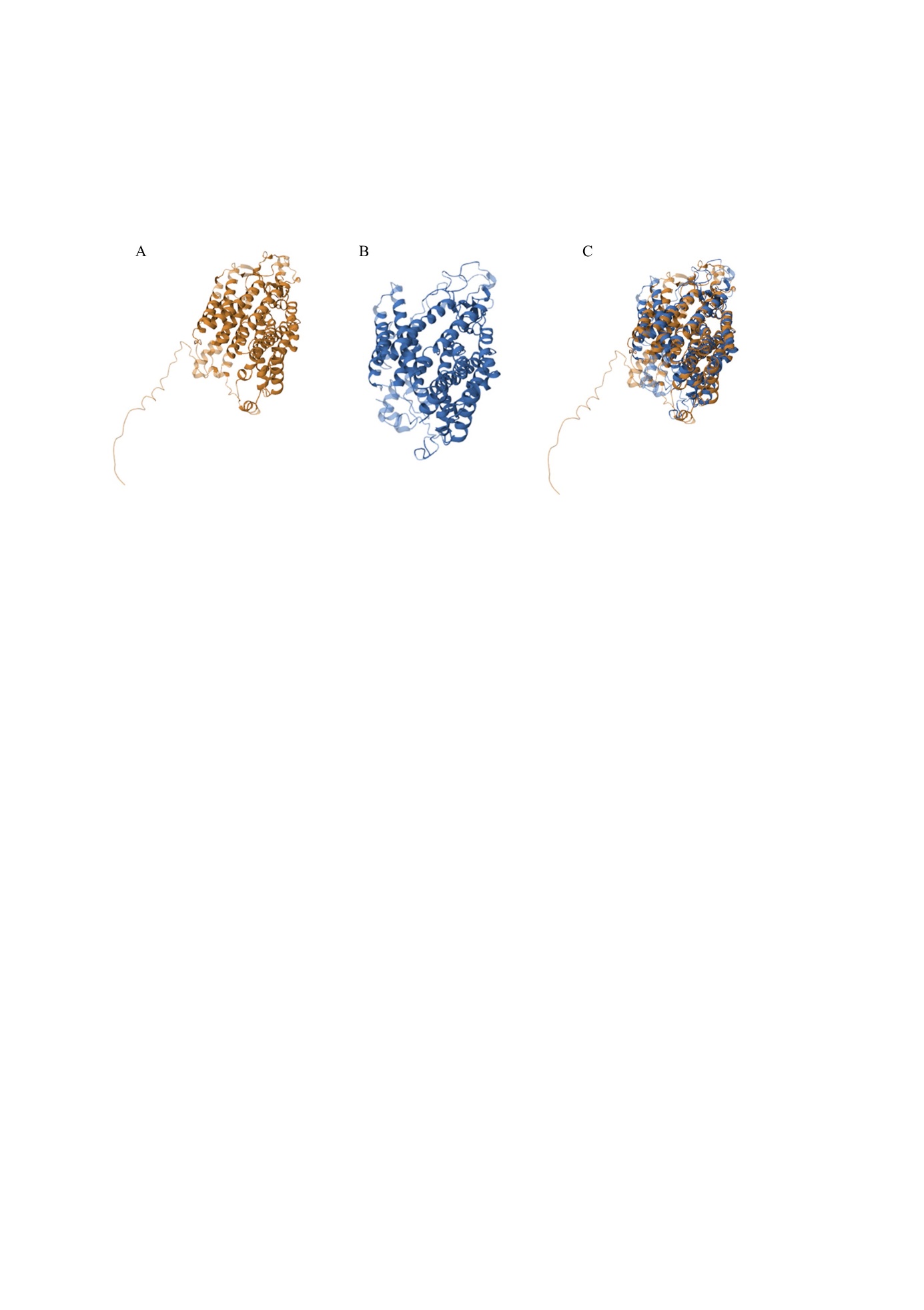


**Figure S15.** Effect of SLC22A5:NM_003060.4:c.254_265del:p.Arg85_Ile89delinsLeu variant on 3D structure of the SLC22A5 protein. A. Wildtype, B. Mutant and C. Merge.


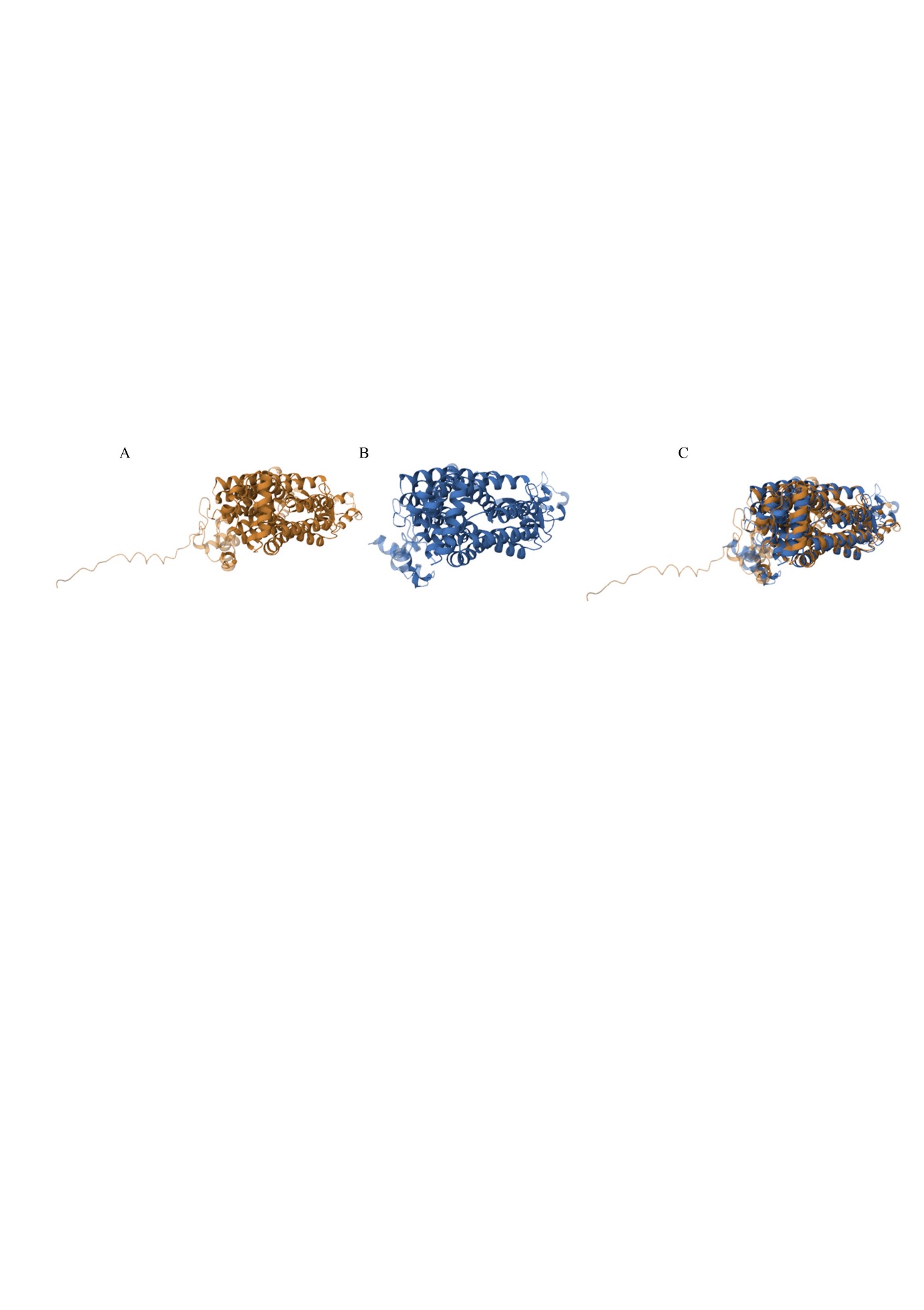


**Figure S16.** Effect of SLC22A5:NM_003060.4:c.1519_1524del:p.Phe508_Leu509del variant on 3D structure of the SLC22A5 protein. A. Wildtype, B. Mutant and C. Merge.


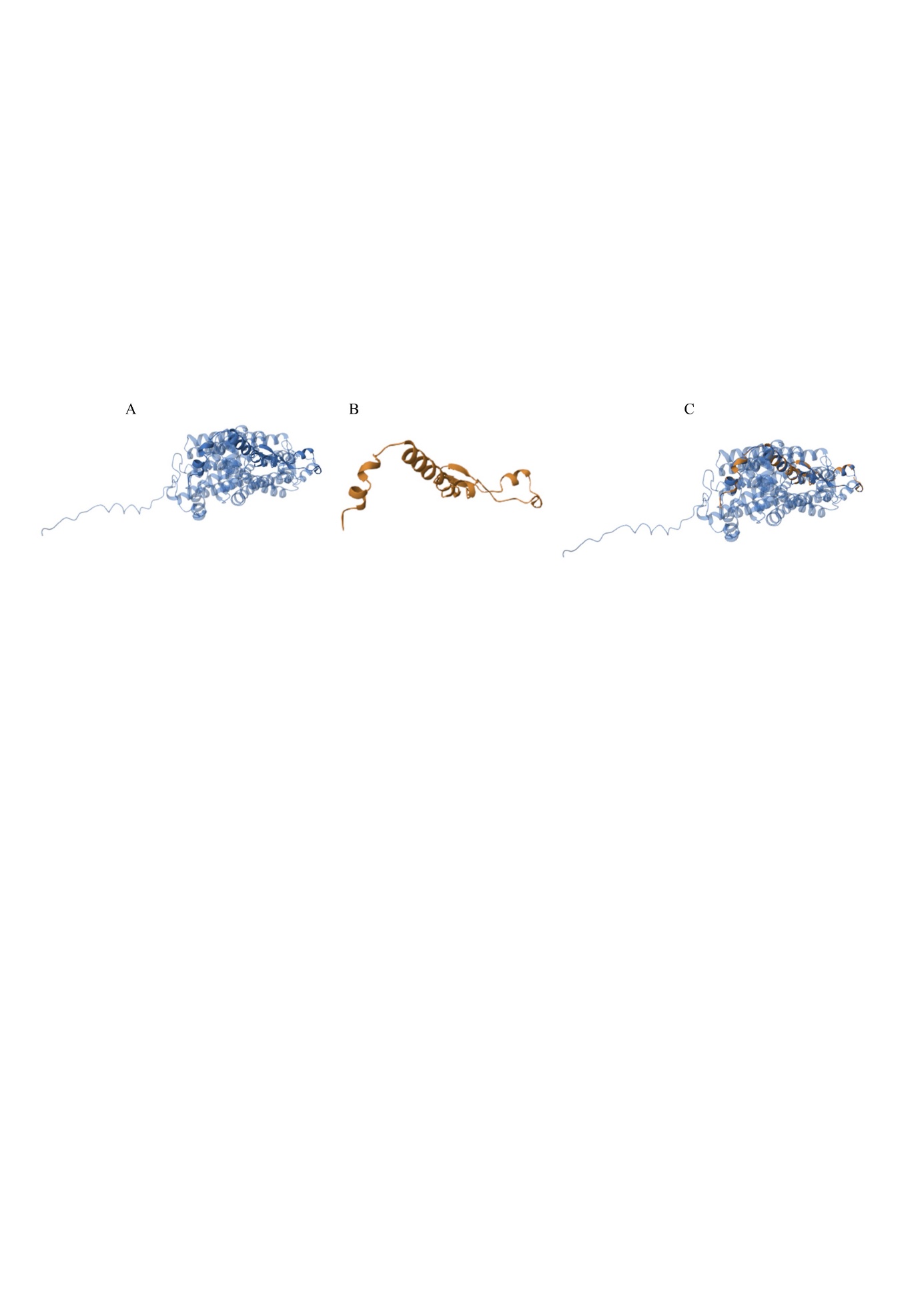


**Figure S17.** Effect of SLC22A5:NM_003060.4:c.217del:p.Asp73Thrfs*57 variant on 3D structure of the SLC22A5 protein. A. Wildtype, B. Mutant and C. Merge.


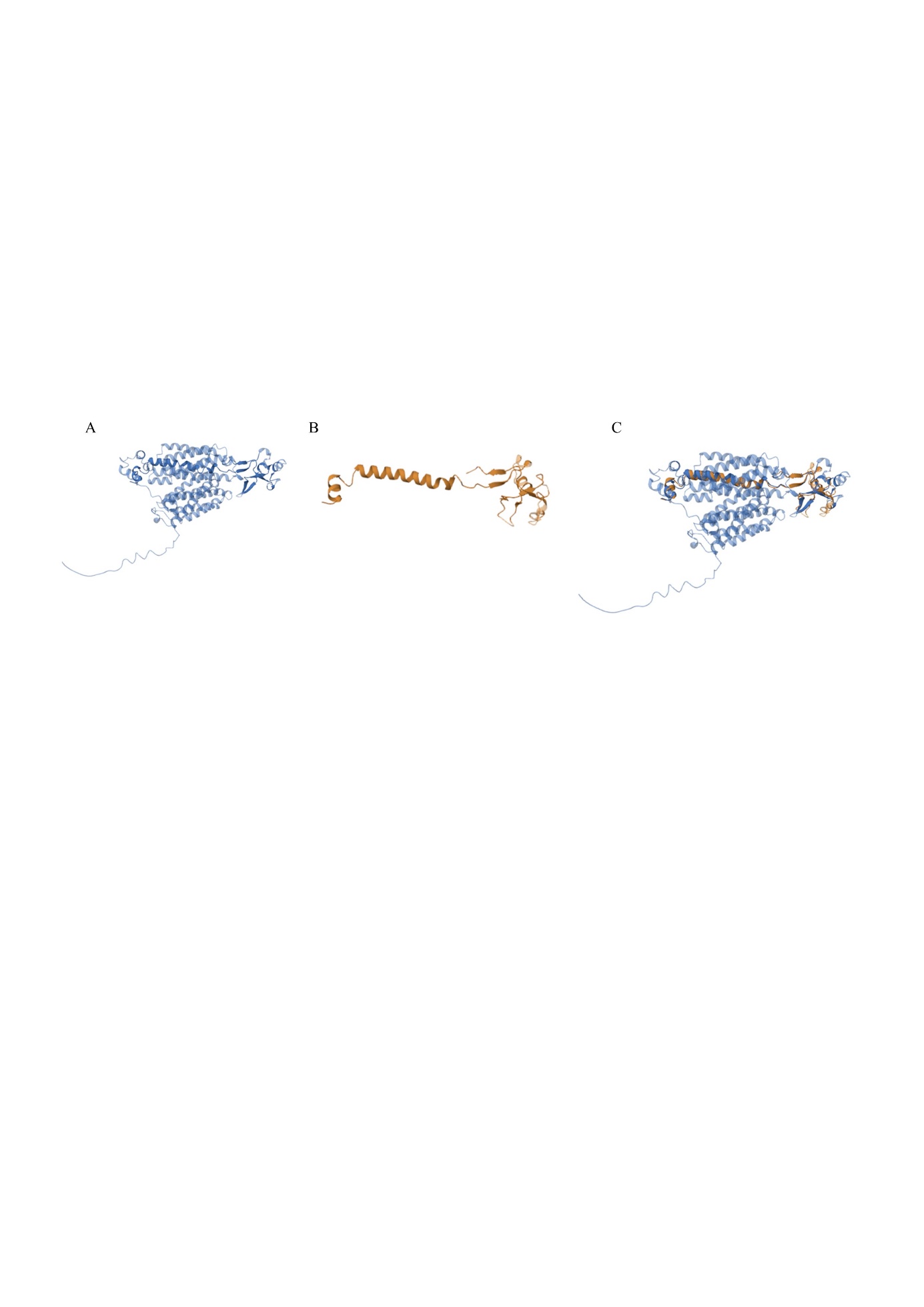


**Figure S18.** Effect of SLC22A5:NM_003060.4:c.249_250insACCGGCTCGCC:p.Tyr84Thrfs*50 variant on 3D structure of the SLC22A5 protein. A. Wildtype, B. Mutant and C. Merge.
